# Coupling Root Diameter With Rooting Depth to Reveal the Heterogeneous Assembly of Root-Associated Bacterial Communities in Soybean

**DOI:** 10.3389/fmicb.2021.783563

**Published:** 2021-12-03

**Authors:** Wen Luo, Xiaoyu Zai, Jieyu Sun, Da Li, Yuanli Li, Guoqiang Li, Gehong Wei, Weimin Chen

**Affiliations:** State Key Laboratory of Crop Stress Biology in Arid Areas, College of Life Sciences, Northwest A&F University, Xianyang, China

**Keywords:** rhizocompartment, rhizosphere microbiota, legume plant, shallow root system, root traits

## Abstract

Root diameter and rooting depth lead to morphological and architectural heterogeneity of plant roots; however, little is known about their effects on root-associated microbial communities. Bacterial community assembly was explored across 156 samples from three rhizocompartments (the rhizosphere, rhizoplane, and endosphere) for different diameters (0.0–0.5 mm, 0.5–1.0 mm, 1.0–2.0 mm, and>2.0 mm) and depths (0–5 cm, 5–10 cm, 10–15 cm, and 15–20 cm) of soybean [*Glycine max* (L.) Merrill] root systems. The microbial communities of all samples were analyzed using amplicon sequencing of bacterial 16S rRNA genes. The results showed that root diameter significantly affected the rhizosphere and endosphere bacterial communities, while rooting depth significantly influenced the rhizosphere and rhizoplane bacterial communities. The bacterial alpha diversity decreased with increasing root diameter in all three rhizocompartments, and the diversity increased with increasing rooting depth only in the rhizoplane. Clearly, the hierarchical enrichment process of the bacterial community showed a change from the rhizosphere to the rhizoplane to the endosphere, and the bacterial enrichment was higher in thinner or deeper roots (except for the roots at a depth of 15–20 cm). Network analysis indicated that thinner or deeper roots led to higher bacterial network complexity. The core and keystone taxa associated with the specific root diameter class and rooting depth class harbored specific adaptation or selection strategies. Root diameter and rooting depth together affected the root-associated bacterial assembly and network complexity in the root system. Linking root traits to microbiota may enhance our understanding of plant root-microbe interactions and their role in developing environmentally resilient root ecosystems.

## Introduction

Plant roots harbor a high diversity of microbiota in various root habitats, including the rhizosphere, rhizoplane, and endosphere (Edwards et al., 2015). The interactions between the root-associated microbiota and plants range from parasitism (detrimental to the host) to mutualism (mutually beneficial), and their outcome can be pivotal for plant performance (Berendsen et al., 2012; Bulgarelli et al., 2012). Studies have reported that plant root-associated microbes provide a plethora of functional capacities and influence plants by modulating root architecture, nutrient acquisition, or resistance to biotic and/or abiotic stressors (Panke-Buisse et al., 2014; Reinhold-Hurek et al., 2015; Vandenkoornhuyse et al., 2015; Verbon and Liberman, 2016). Recent studies further indicated that root phenotypic traits could lead to variations in microbial communities (Saleem et al., 2018; Pervaiz et al., 2020). Investigating the relationships between root traits and root-associated microbiota could improve the fundamental understanding of root-associated microbial assembly processes and associations between microorganisms and plants.

Root diameter and rooting depth are important root morphological and architectural traits that determine the spatial configuration of the root system, and roots of different diameters or depths could potentially differ in their physical, biochemical, and functional traits (Bardgett et al., 2014; Ma et al., 2018b; Liu et al., 2019; Germon et al., 2020; Pervaiz et al., 2020). Fine roots (root diameter<2 mm) enable plants to efficiently leverage photosynthetic carbon for exploring resource-rich soil patches (Hoeppner and Dukes, 2012), while coarse roots (root diameter>2 mm) mainly support the fine root network and plant structure to deliver and store nutrients (Ma et al., 2018a). Deeper roots enable the acquisition of water under drought (Debinski et al., 2010; Hoeppner and Dukes, 2012). In general, plant roots distribute more roots in superficial soil layers than in subsoil layers, so root length densities and surface area densities decrease with increasing soil depth (Hati et al., 2006). Microbes can affect plant growth by modifying root development and traits (Verbon and Liberman, 2016). Linking microbial communities to the root categories may increase our understanding of the microbial communities and their functions and variations in the context of soil and plant health.

To date, a few studies have investigated the obvious impact of root heterogeneities on microbial communities (Perez-Jaramillo et al., 2017; Yahara et al., 2019; Gray and Kernaghan, 2020). The fine, secondary, and primary roots of tobacco plants harbor distinct microbial communities (Saleem et al., 2016, 2018). Wang et al. (2017a) indicated that low root orders of poplar trees harbored higher bacterial diversity in the rhizosphere than high root orders. Yang et al. (2016) also reported a significant difference in microbial communities between shallow and deep roots of Jerusalem artichokes. However, the variation in the microbial communities along the root diameter and rooting depth gradients was not well profiled together.

The plant root microbiome is predominantly assembled from the surrounding soil. The assembly patterns of root-associated microbial communities have been shown with a three-step enrichment model from the rhizosphere soil to the root rhizoplane and to the plant endosphere (Lundberg et al., 2012; Edwards et al., 2015; Zhang et al., 2017). Briefly, a specialized community in the bulk soil is enriched toward the roots due to general nutrient gradients imposed by the roots and forms the rhizosphere microbiome. Then, a more specialized microbial community is further enriched on the rhizoplane following close microbe-host interactions. Finally, certain microbiota enter and inhabit the root interior, forming the endosphere microbiome. The successful colonization of microbes requires the capability to overcome the host immune system and abiotic stresses (Vorholt, 2012; Bulgarelli et al., 2013). The assembly patterns have been mainly studied among plant species or genotypes (Bulgarelli et al., 2012; Mendes et al., 2014; Xiong et al., 2021). Dissecting the enrichment and filtration patterns from rhizosphere to rhizoplane to endosphere can give us a more comprehensive insight into the microbial assembly process along the root diameter and root depth.

Soybean [*Glycine max* (L.) Merrill] is one of the most widely grown legume crops. Soybean roots with a taproot system are mainly distributed in the topsoil layer (0–20 cm depth) and have strong plasticity under different nutrient stresses and agricultural practices (Benjamin and Nielsen, 2006; Meister et al., 2014; Li et al., 2017). Although the root-associated microbiomes of soybean have been well studied (Madhaiyan et al., 2014; Sugiyama et al., 2014; Zhang et al., 2018), little is currently known about the relationships between the microbiomes and root diameter or rooting depth. In the present study, we conducted a detailed examination of the rhizosphere, rhizoplane, and endosphere bacterial communities along root diameter and rooting depth gradients using Illumina sequencing of the 16S rRNA gene. The objectives were to answer the following questions. (i) Do the root-associated bacterial communities exhibit different diversities along the root diameter or rooting depth gradients?, (ii) do root diameter and rooting depth together affect the enrichment of bacterial communities from the root rhizosphere to the endosphere?, and (iii) what associations exist among bacterial taxa in roots with different diameters or depths? Addressing these questions is essential for understanding the interactions between the microbial community and plant root systems, and we expect to provide a root diameter-dependent and rooting depth-dependent model for analyzing the microbial community structure in root systems.

## Materials and Methods

### Experimental Site and Sampling Methods

Sampling was conducted in September 2018 at the podding stage of soybean in an agricultural experimental field (34°18' N, 108°6' E) on the Guanzhong Plain of northwestern China, with an average yearly temperature of 14.6°C and a mean annual precipitation of 590.3 mm. Soil in the experimental area is classified as a Fimic Anthrosol, and the topsoil texture classification is loam (Harmonized World Soil Database, FAO Soil Taxonomic system). The experimental field was previously planted with soybeans. The soybean [*Glycine max* (L.) Merrill, Zhonghuang 13] studied in this experiment is a widely grown soybean cultivar in China. The intrarow and interrow plant spacing used was 40 cm. Conventional management practices using hand hoe with no fertilizer and pesticide inputs represented farmers’ normal practice for soybean production in the area. The plots were ploughed in June before cultivation. Weeds were removed with hand hoes as needed during soybean growth development. Appropriate irrigation was carried out at the branching stage and flowering stage of soybean. The amount of irrigation was set based on the accumulated experience of the local farmers during agricultural production and the precipitation in the study area.

Three random subplots (approximately 5×5 m each) were chosen, and the subplots were spaced 5 m apart to preclude interactions. Each soybean root was collected within a 20-cm distance from the main stem and within a 20-cm vertical profile depth according to the main distribution area of a soybean root system in our study. For each plot, a group of twenty fresh roots was collected from four soil layers (Layer I, 0–5 cm; Layer II, 5–10 cm; Layer III, 10–15 cm; and Layer IV, 15–20 cm). Meanwhile, a group of five bulk soil samples away from plant roots was also collected from four soil layers. The roots and soil from the same soil layer were mixed and then sealed in zip-lock bags for transport to the laboratory in an ice box. Subsequently, the roots of the same soil layer were divided into four classes based on root diameter (Root I, 0.0–0.5 mm; Root II, 0.5–1.0 mm; Root III, 1.0–2.0 mm; and Root IV, >2.0 mm) using a graduated ruler (0.1mm resolution).

Furthermore, the roots of each diameter class were used for the sample collection of three rhizocompartment fractions (rhizosphere, rhizoplane, and endosphere) according to a previously described method (Edwards et al., 2015). Briefly, the roots were placed in a sterile tube with sterile phosphate buffer and then vortexed to collect the soil adhering to roots as the rhizosphere compartment. The roots in the tube were sonicated for 30 s each (time interval 30 s) at 50 Hz to strip the rhizoplane microbes from the root surface. The roots were then removed, and the liquid PBS (phosphate buffer solution) fraction was centrifuged and retained as the rhizoplane compartment. The sonicated roots were then placed in a new tube and sonicated twice more, and these roots were retained as the endosphere compartment. All rhizocompartment samples were stored at −80°C for DNA extraction. Bulk soils were divided into two subsamples. One bulk soil subsample was stored at −80°C for subsequent DNA extraction. The remaining bulk soil subsample was used to analyze the soil physicochemical properties. Finally, a total of 156 samples (four root diameters × four rooting depths × three rhizocompartments × three replicates + bulk soil × four soil layers × three replicates) were used for DNA extraction and PCR amplification of the V4-V5 regions of the 16S rRNA gene and subsequent high-throughput sequencing.

### Soil Physical and Chemical Property Analyses

Soil pH, soil water content (SWC), total organic carbon (TOC), total nitrogen (TN), total phosphorus (TP), total potassium (TK), nitrate nitrogen (NO_3_^−^-N), ammonium nitrogen (NH_4_^+^-N), available phosphorus (AP), and available potassium (AK) according to routine methods (Bao, 2000). Soil pH was determined with a fresh soil to water ratio of 1:5 using a pH meter (Thermo Fisher Scientific, MA, United States). SWC was measured by the oven-drying method. TOC was determined by applying a traditional dichromate oxidation titration. TN was quantified following the Kjeldahl method. The NH_4_^+^-N and NO_3_^−^-N were determined by extraction with 1M KCl, steam distillation, and titration following the alkaline diffusion method. TP was extracted using 1 M HCl after ignition, and AP were extracted using 0.5 M NaHCO_3_, and then, they were measured following the Mo-Sb colorimetric method. AK extracted by 1 M CH_3_COONH_4_ and TK were measured using the flame photometry method.

### DNA Extraction and High-Throughput Sequencing

Genomic DNA was extracted from bulk soil (0.5 g) and rhizosphere soil (0.5 g) using a FastDNA SPIN Kit for soil (MP Biomedicals, Solon, OH, United States) according to the manufacturer’s protocols. Due to the lower amount of rhizoplane samples, they were resuspended in 100 μl of PBS. Then, the genomic DNA of the root rhizoplane liquid samples was also extracted using the FastDNA SPIN Kit for soil. The extraction of the root samples (0.5 g) was performed using an Invisorb Spin Plant Mini Kit (Stratec Biomedical AG, Birkenfeld, Germany). The V4-V5 region of the 16S rRNA gene was amplified using the primer pair F515 (5'-GTGCCAGCMGCCGCGGTAA-3') and R907 (5'-CCGTCAATTCCTTTGAGTTT-3'; Edwards et al., 2015). Single-end high-throughput sequencing of the purified PCR products (400–450 bp) was performed on an IonS5TMXL platform (Thermo Fisher) at Novogene Bioinformatics Technology Co., Ltd. (Beijing, China). The acquired reads were quality-filtered using Cutadapt (Martin, 2011) and Vsearch (Rognes et al., 2016). Then, the sequences were clustered into operational taxonomic units (OTUs) based on >97% similarity using Uparse (Edgar, 2013). The representative sequences were further annotated against the SSU rRNA database of the SILVA 132 Database (http://www.arbsilva.de/) using Mothur (Schloss et al. 2009). Abundance data of sequences matching chloroplasts and mitochondria were removed from the datasets. To minimize the impact of sequencing artifacts, singletons were removed from the datasets.

### Statistical Analysis

The statistical analyses were performed using R software 3.4.4 (http://www.r-project.org). Alpha diversity indices were calculated using the package “vegan” (Dixon, 2003). Principal coordinates analysis (PCoA) based on Bray-Curtis distances and constrained analysis of principal coordinates (CAP) based on Bray-Curtis distances were performed to visualize the relationships between samples. Permutational multivariate analysis of variance (PERMANOVA) based on Bray-Curtis distances was performed to determine whether sample classifications contained significant differences in species diversity using the package “vegan.” The “edgeR” package (Robinson et al., 2009) was used to identify the significantly altered OTUs by fitting a negative binomial distribution model to the 6,908 OTUs. The OTU counts from the bulk soil served as a control, and the adjusted *p* value cutoff was 0.05. The ratio of the enriched to depleted index (EDI) was defined to evaluate the bacterial enrichment effect in each rhizocompartment (Xiong et al., 2021). The core taxa associated with each root diameter or rooting depth were identified by canonical discriminant analysis (CDA) using the “candisc” package (Friendly and Fox, 2015).

OTUs with relative abundances higher than 0.1% were selected to construct co-occurrence networks. Spearman’s correlation between two OTUs was considered statistically robust if Spearman’s correlation coefficient (ρ) was >0.6 and the value of *p* was <0.01. To describe the network topology features, a set of measures (number of total nodes, total links, negative links, positive links, average path length, average degree, average clustering coefficient and modularity) were calculated using the “igraph” package (Csardi and Nepusz, 2006). The natural connectivity and average degree after the removal of nodes in the static network were used to estimate the network stability (Peng and Wu, 2016). The networks were visualized using the interactive platform Gephi (Bastian et al., 2009) or Cytoscape (Shannon, 2003). Keystone species were identified according to the betweenness centrality value, which indicates the relevance of a node as capable of holding together communicating nodes (Lupatini et al., 2014). In this study, the OTU with the highest betweenness centrality value was considered to be the keystone taxon.

## Results

### Alpha Diversity and Beta Diversity of Bacterial Community

**Figure 1:**
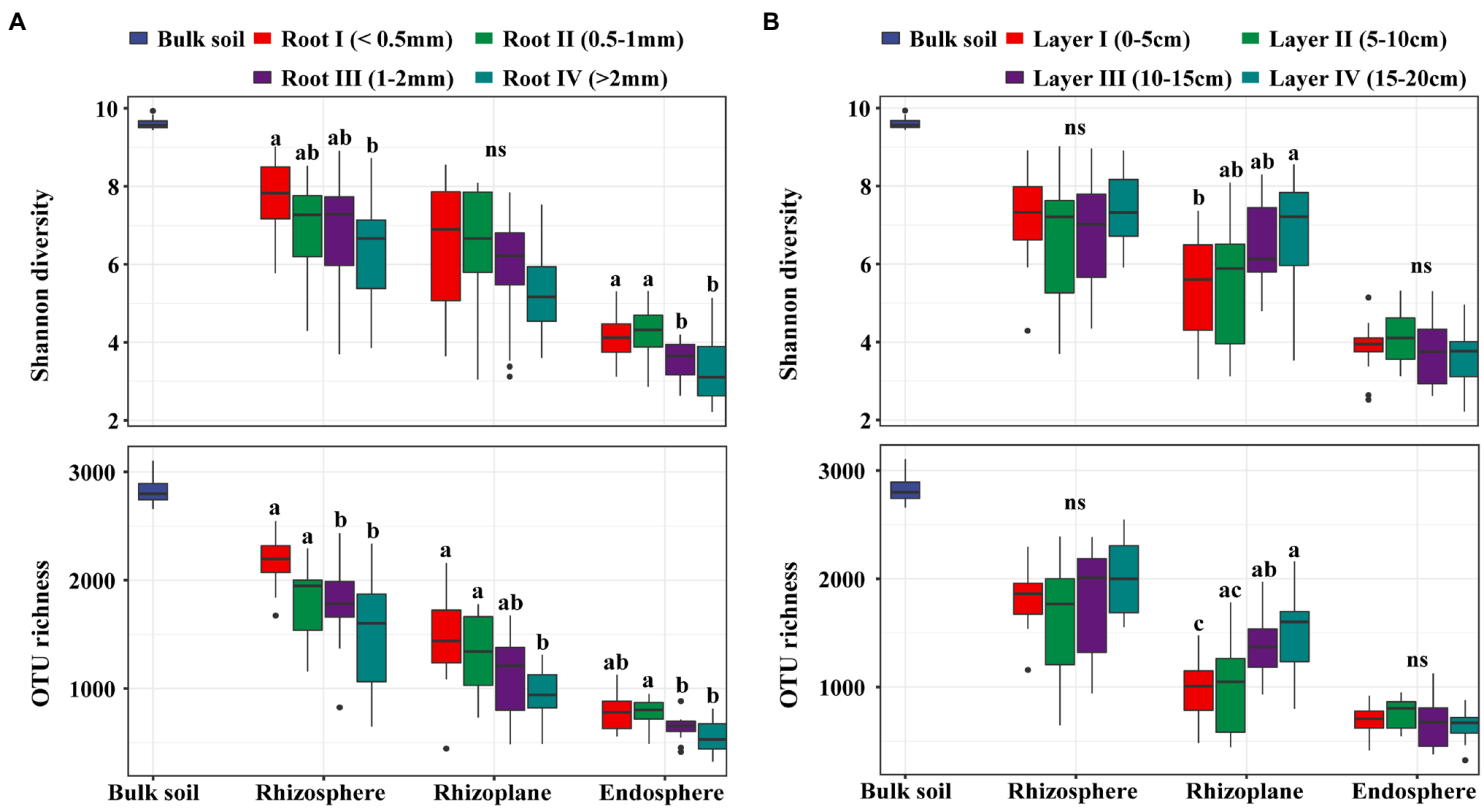
The boxplot of Shannon diversity and OTU richness (number of observed OTUs) of bacterial communities in rhizosphere, rhizoplane and endosphere among different root diameters (**A**; *n*=12) or different rooting depths (**B**; *n*=12). Different letters above the box indicate significant differences (*p*<0.05, “ns” to indicate non-significance, multiple comparison with Kruskal-Wallis tests).

In our study, there was no significant difference in the soil physical and chemical properties among the different soil layers, suggesting that the soil environment was homogeneous across the 0–20 cm layer (Supplementary Table S1). A total of 9,637,423 high-quality sequences from all 156 samples were clustered into 6,908 OTUs and classified into 54 phyla and 651 genera. In general, the bacterial taxon numbers at all levels (from genus to phylum) and the diversity indices were different among the four root diameters and four rooting depths (Supplementary Table S2). Alpha diversities, expressed as the OTU richness (the observed OTUs) and the Shannon diversity (Shannon-Wiener index), were significantly influenced by both root diameter and rooting depth. The bacterial alpha diversity in all three rhizocompartments showed a significantly increasing trend with decreasing root diameter (Figure 1A). The bacterial alpha diversity in the rhizoplane significantly increased with increasing rooting depth (Figure 1B).

**Figure 2:**
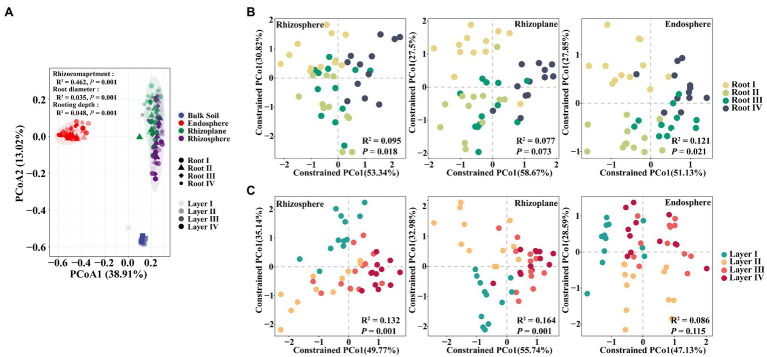
Principal coordinates analysis (PCoA) based on Bray-Curtis distances and constrained analysis of principal coordinates (CAP) based on Bray-Curtis distances of soybean root-associated microbiome. **(A)** PCoA plot showing differences in the beta diversity of all samples. **(B)** CAP plot showing bacterial compositional variation in rhizosphere, rhizoplane or endosphere explained by root diameter when controlling for rooting depth. **(C)** CAP plot showing bacterial compositional variation in rhizosphere, rhizoplane or endosphere explained by rooting depth when controlling for root diameter. Variance values were examined *via* PERMANOVA test, which are shown in each plot.

To further explore the influence of root diameter and rooting depth on the entire root-associated microbiota structure, we performed PCoA, CAP, and PERMANOVA. The PERMANOVA results showed that the largest source of variation in the microbial communities was the rhizocompartment based on a Bray-Curtis distance metric (*R*^2^=0.462, *p*=0.001). Small but significant effects were also explained by the root diameter (*R*^2^=0.035, *p*=0.001) and rooting depth (*R*^2^=0.048. *p*=0.001). The PCoA plot (Figure 2A) confirmed that the microbiomes in different rhizocompartments clearly separated from each other; however, the clustering patterns of the microbiomes among different root diameters or rooting depths were not evident. The CAP results further showed that the Root I group, Root IV group, and the Root II and Root III groups were clearly separated (Supplementary Figure S1). Similar clustering patterns also occurred in the rhizosphere (*R*^2^=0.095, *p*=0.018) and endosphere (*R*^2^=0.121, *p*=0.021; Figure 2B). Among rooting depths, the Layer I group, Layer II group, and the Layer II and Layer IV groups were separated. Clustering patterns occurred in the rhizosphere (*R*^2^=0.132, *p*=0.001) and rhizoplane (*R*^2^=0.164, *p*=0.001; Figure 2C). Pairwise comparisons of the bacterial community between root diameters or rooting depths based on PERMANOVA further confirmed these results (Supplementary Table S4). In summary, the root diameter and rooting depth significantly influenced the composition of the root-associated bacterial community.

### Distribution and Enrichment Process of the Bacterial Community

**Figure 3:**
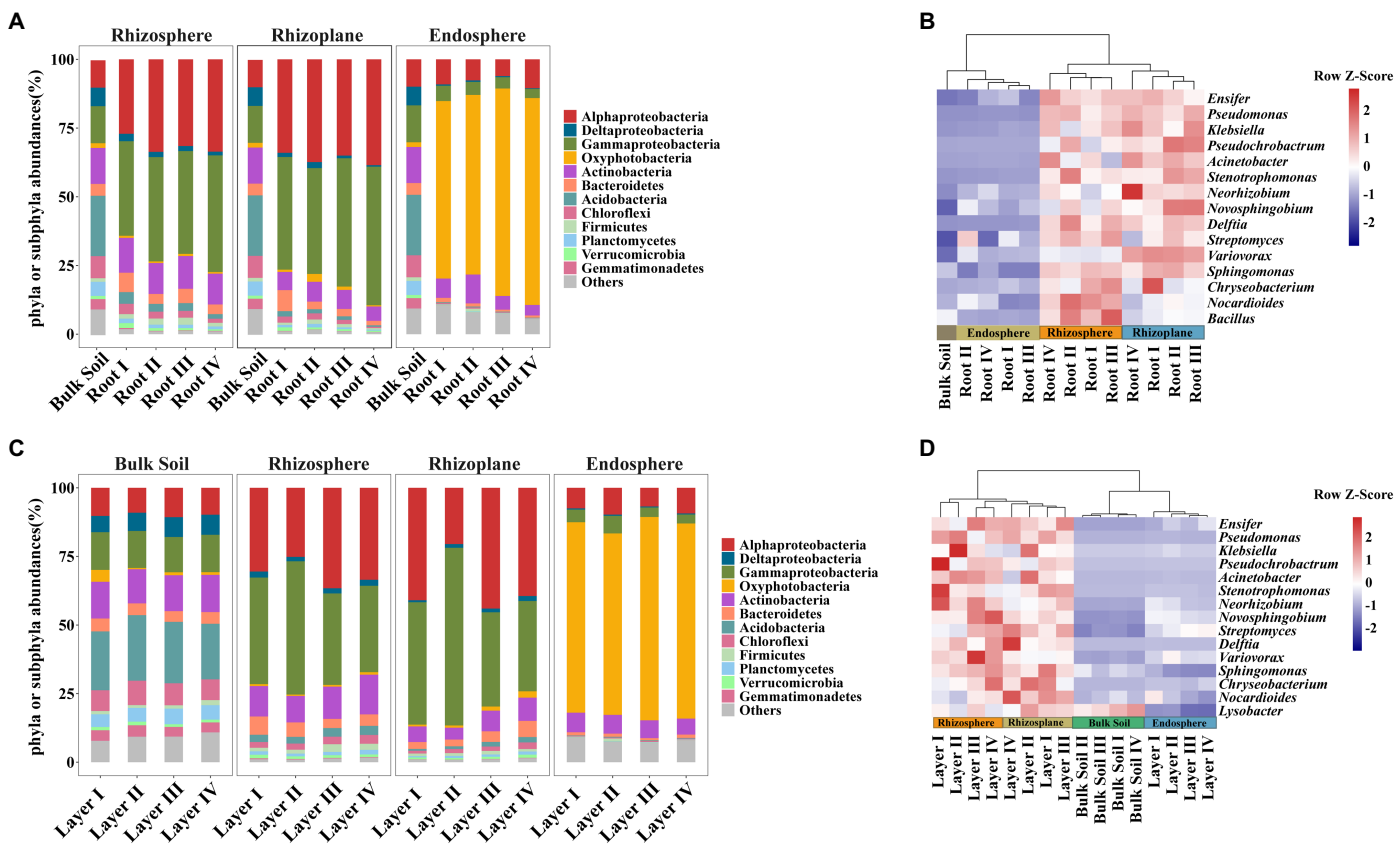
Bacterial community composition plots in different root diameters and rooting depths. **(A,C)** Bar chart showing the relative abundance of dominant bacterial phyla or subphyla (top 10). **(B,D)** Heatmap showing the distribution of bacterial genus level (top 15). Heatmap color (blue to dark red) displays the row scaled relative abundance of each taxon across all samples.

Taxonomic differences between the root classes based on the dominant phyla or subphyla (top 10 abundance) and genera (top 15 abundance) are displayed in bar plots and pheatmaps (Figure 3). The phyla Deltaproteobacteria, Bacteroidetes, Acidobacteria, and Chloroflexi significantly varied among root diameters (Supplementary Table S5). Meanwhile, the genus *Neorhizobium* was more abundant in the rhizoplane of Root III and Root IV, and the genus *Streptomyces* was more abundant in the endosphere of Root I and Root II (Figure 3B and Supplementary Table S6). The phyla Alphaproteobacteria, Deltaproteobacteria, Gammaproteobacteria, Oxyphotobacteria, Actinobacteria, Acidobacteria, and Planctomycetes in both the rhizosphere and rhizoplane significantly varied among rooting depths, while Oxyphotobacteria and Gammaproteobacteria significantly varied in the endosphere (Supplementary Table S5). The genus *Klebsiella* was more abundant in the rhizosphere of Layer I, Layer II, and Layer III, *Novosphingobium*, *Variovorax*, and *Delftia* were more abundant in the rhizoplane of Layer III and Layer IV, and *Pseudomonas* and *Pseudochrobactrum* were more abundant in the endosphere of Layer I (Figure 3D and Supplementary Table S6). In addition, the heatmap plot showed a strong clustering of different root diameters or rooting depths (Figures 3B,D), which was similar to the CAP results. These results indicated the different distribution patterns of dominant species abundance among root diameters or rooting depths.

**Figure 4:**
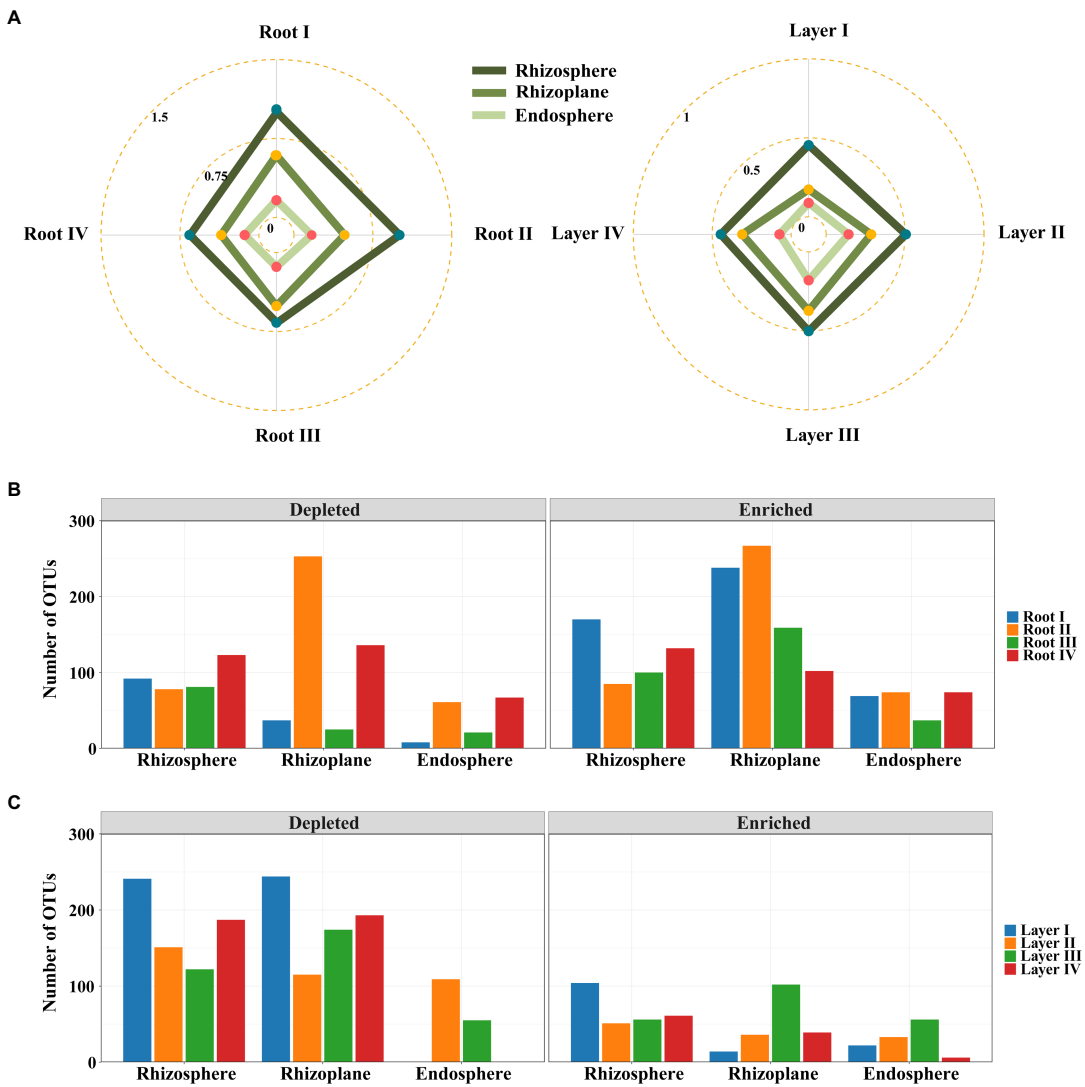
Enrichment and filtration of bacterial communities within different root diameters and rooting depths. **(A)** Radar plots of the ratios of statistically significantly enriched OTUs to the depleted OTUs with root diameter and rooting depth. Bar plots of the specifically enriched and depleted OTUs of the microbiota colonized in rhizocompartments of shared among root diameters **(B)** or rooting depths **(C)**.

Differential abundance analysis was used to explore the changes in the significantly enriched and filtered OTUs among the different root diameters or rooting depths. The EDI value (the ratio of significantly enriched OTUs to depleted OTUs) was used to evaluate the species enrichment and filtration processes from the bulk soil to other root-associated niches. The EDI value decreased in the order of rhizosphere > rhizoplane > endosphere for all roots (Figure 4A; Supplementary Figure S4), suggesting that the root-associated bacterial communities were mainly derived from bulk soils and were gradually enriched and filtered in different rhizocompartments by a hierarchical process. Within each rhizocompartment, the EDI values were clearly different among root diameters or rooting depths (Figure 4A; Supplementary Figure S4). Generally, the EDI value had an increasing trend with decreasing root diameter and increasing rooting depth (except Layer IV). The roots of Root I and Layer III showed the highest EDI values across the three rhizocompartments, suggesting the largest enrichment effect of root-associated bacterial communities. These results suggested that root diameter and rooting depth significantly influenced the hierarchical process of bacterial enrichment and filtration in the root system.

Irrespective of root diameters or rooting depths, the overlaps of enriched OTUs were mainly from Proteobacteria, Actinobacteria, and Bacteroidetes in the three rhizocompartments. In the rhizosphere and rhizoplane, the enriched genera mainly belonged to *Ensifer*, *Pseudomonas*, *Pseudochrobactrum*, *Klebsiella*, *Acinetobacter*, *Stenotrophomonas*, and *Delftia*, while the enriched genera mainly belonged to *Ensifer*, *Streptomyces*, *Novosphingobium*, *Neorhizobium*, *Variovorax*, *Phytohabitans*, and *Actinoplanes* in the endosphere (Supplementary Figures S2, S3). Among them, *Ensifer* was strongly enriched in all rhizocompartments of all root classes. Almost all of these genera were members of the dominant genera (Figures 3B,D). In addition, Root I had the highest number of specifically enriched OTUs in the rhizosphere, and Root IV had the highest number of specifically enriched OTUs in the rhizoplane and endosphere (Figure 4B; Supplementary Figure S4). Among rooting depths, Layer I had the highest number of specifically enriched OTUs in the rhizosphere, and Layer III had the highest number of specifically enriched OTUs in the rhizoplane and endosphere (Figure 4C; Supplementary Figure S4). The five specifically enriched and dominant genera in each root diameter and rooting depth (Supplementary Figures S5, S6) further showed distinct enrichment functions in different root classes.

### Variation of the Root-Associated Core Bacterial Community

The root core microbiome (the broadly distributed microbial taxa in the root system) was identified as the bacterial taxa present in at least 95% of the samples (n=144). The 130 core taxa identified represented only 13.49% of the total number of OTUs, but these taxa accounted for the majority of the reads (57.11%). The relative abundance of the core taxa significantly varied among root diameters in the endosphere and among rooting depths in the rhizoplane and rhizosphere (Supplementary Figure S7). The significant effects were further confirmed in the endosphere (root diameter, *R*^2^=0.15, *p*=0.031), rhizoplane (rooting depth, *R*^2^=0.16, *p*=0.001), and rhizosphere (rooting depth, *R*^2^=0.21, *p*=0.001) using PERMANOVA.

**Figure 5:**
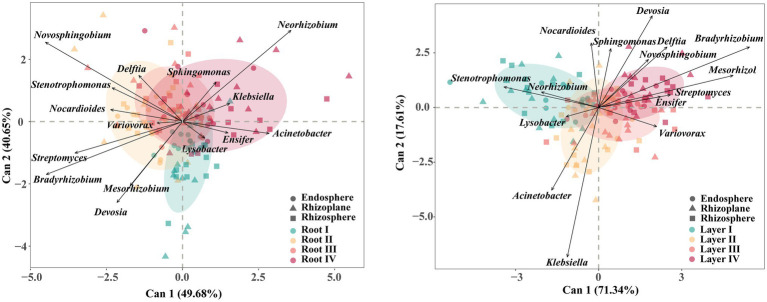
Canonical discriminant analysis (CDA) plot based on the predominant (top 15 abundance) core bacterial taxa at genus level. Arrows represent the degree of correlation between each taxon and each root diameter or rooting depth. Circles represent the canonical group means and 85% confidence interval for each root class.

The core taxa were mainly members of the phyla Proteobacteria, Actinobacteria, Acidobacteria, and Gemmatimonadetes. The CDA results of the 15 most abundant core genera revealed differences in their associations with different root diameters and rooting depths (Figure 5; Supplementary Figure S8). For example, *Novosphingobium* and *Bradyrhizobium* were associated with the Root II group, and *Neorhizobium* was associated with the Root IV group. *Stenotrophomonas*, *Klebsiella*, *Variovorax*, and *Bradyrhizobium* were associated with the Layer I, Layer II, Layer III, and Layer IV groups, respectively.

### Co-occurrence Network of the Root-Associated Bacterial Communities

**Figure 6:**
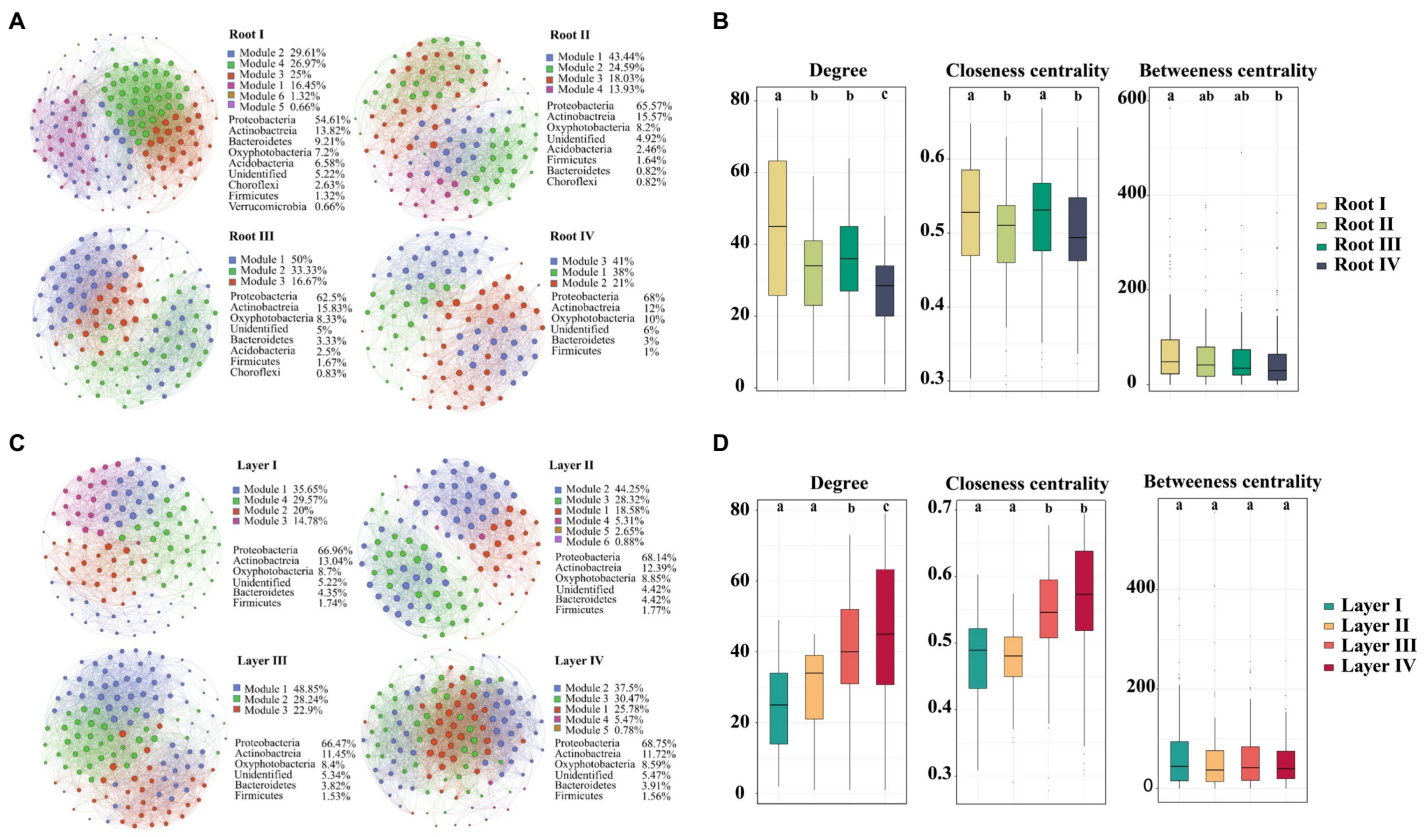
An operational taxonomic unit (OTU) network map showing the composition and correlation of the OTUs in different root diameters **(A)** and rooting depths **(C)**. Network connections indicate a strong (Spearman’s ρ>0.6) and significant (*p*<0.01) correlation. The size and color shade of each node is proportional to the number of connections (degree), and the thickness of each connection between two nodes (edge) is proportional to the value of Spearman’s correlation coefficients. Unique node-level topological features of bacteria of different root diameters (B) and rooting depths **(D)**, specifically the degree, betweenness, closeness. Different letters above the box indicate significant differences (*p*<0.05, multiple comparison with Kruskal-Wallis test).

To investigate the variation in interrelationships between root-associated bacterial taxa along root diameter and rooting depth, network analysis was performed (Figure 6). The topological features of the networks were calculated at the unique node (OTU) level (Table 1). The average clustering coefficient and average path length in these empirical networks were greater than those in random networks, suggesting that the empirical networks had the properties of “small worlds” and nonrandom co-occurrence patterns. The topological structure of bacterial networks of different root diameters and rooting depths was different from those of the whole root system and bulk soil networks (Table 1; Supplementary Figure S9). In addition, significant variations in the topological structure of networks occurred along the root diameter and rooting depth gradients (Figures 6B,D; Table 1). The network complexity (a higher average degree representing a greater network complexity) decreased in the order of Root I>Root II/Root III>Root IV and of Layer IV>Layer III>Layer II>Layer I (Table 1). The ratio of negative links to positive links (N/P) and modularity values (MD) exhibited similar trends in the bacterial networks. Natural connectivity represents the stability of network topology against random destruction or intentional attacks. The values of natural connectivity in all root networks decreased sharply when more network nodes were randomly removed. The natural connectivity value decreased in the order of Root I>Root II/Root III>Root IV and the order of Layer IV>Layer III/Layer II>Layer I (Supplementary Figure S10).

**Table 1:** Topological features the bacterial networks of different root diameters and rooting depths.

	Empirical networks									Random networks	
	Nodes	Links	PL	NL	N/P	MD	ACC	APL	AD	ACC	APL
Root I	152	3,307	2,863	444	0.16	0.32	0.71	1.99	43.51	0.288±0.002	1.712±0.001
Root II	122	1905	1,507	398	0.26	0.57	0.69	2.04	31.23	0.258±0.003	1.742±0.001
Root III	120	2027	1,655	372	0.22	0.44	0.70	1.97	33.78	0.284±0.003	1.716±0.001
Root IV	100	1,292	953	339	0.36	0.75	0.70	2.06	25.84	0.261±0.004	1.740±0.001
Layer I	115	1,392	997	395	0.40	0.93	0.594	2.16	24.21	0.212±0.004	1.792±0.001
Layer II	113	1,659	1,249	410	0.33	0.66	0.734	2.14	29.36	0.262±0.003	1.738±0.001
Layer III	131	2,557	2,126	431	0.20	0.43	0.675	1.89	39.04	0.300±0.002	1.700±0.001
Layer IV	128	2,820	2,319	501	0.22	0.32	0.694	1.84	44.06	0.347±0.002	1.653±0.001

Positive links (PL), negative links (NL), the ratio of negative links to positive links (N/P), modularity (MD), average clustering coefficient (ACC), average path length (APL), average degree (AD).

The taxa composition of the networks clearly varied among root diameters or rooting depths. Of the 184 and 178 distinct OTUs in the networks of different root diameters and rooting depths, only 71 and 73 nodes were shared, respectively (Supplementary FigureS11). Proteobacteria was the primary member of networks of different root classes (root diameters, 54.61–68% of the total nodes; rooting depths, 66.47–68.75%; Figures 6A,B). When the distribution of nodes was modularized, the networks of the root diameters or rooting depths were mainly grouped into three to six modules (Figures 6A,C), and the composition of the modules varied greatly among the root diameters or rooting depths (Supplementary Figure S12). The keystone taxa with the highest betweenness centrality value were members of *Bacillus*, *Ramlibacter*, *Ensifer*, and *Sphingomonas* in the Root I, Root II, Root III, and Root IV groups, respectively. The keystone taxa in the rooting depth networks were members of *Actinoplanes*, *Arthrobacter*, *Pseudomonas*, and *Ensifer* in the Layer I, Layer II, Layer III, and Layer IV groups, respectively.

## Discussion

### Effect of Root Diameter and Rooting Depth on the Composition of Root-Associated Bacterial Communities

Our results based on the complete dataset showed that the rhizocompartment niche considerably influenced the bacterial community assembly (Figure 2). Some studies on the root microbiome of rice, poplar trees, and soybean also indicated that different root niches harbored distinct microbial communities (Edwards et al., 2015; Beckers et al., 2017; Xiao et al., 2017). Notably, root diameter and rooting depth had significant impacts on the root-associated bacterial community, although the effects were smaller in comparison with the rhizocompartment (Figure 2). Together, these results suggest that root diameter and rooting depth can influence the roots, selecting niche-compliant microbes to occupy various rhizocompartments. Previous studies also indicated differentiation of rhizosphere or endosphere bacterial communities between fine and coarse roots (Saleem et al., 2016; Perez-Jaramillo et al., 2017; Wang et al., 2017a) or shallow and deep roots (Yang et al., 2016). Our study further showed that the rhizosphere microbiome was affected by both root diameter and rooting depth, while the rhizoplane and endosphere microbiomes were mainly affected by rooting depth and root diameter, respectively (Figure 2). These results indicated that root diameter and rooting depth jointly determined the selection of the root-associated microbiome.

### Differences in the Bacterial Enrichment Processes Along the Root Diameter and Rooting Depth Gradients

Our results showed that regardless of how the root diameter and rooting depth changed, there was a hierarchical enrichment effect for microbes in the roots from the rhizosphere to the rhizoplane to the endosphere (Figure 4). Such an enrichment effect has been previously reported in the roots of soybean, rice, and Arabidopsis (Lundberg et al., 2012; Edwards et al., 2015; Xiao et al., 2017). We further found that the hierarchical enrichment of bacterial communities varied along the root diameter and rooting depth gradients. The thinner roots (smaller root diameter) and deeper roots displayed higher enrichment effects for the bacterial communities than other root classes. This might be due to a possible functional specialization of these roots to absorb nutrients and water by a large microbial community (Germon et al., 2020). Previous studies indicated that thinner plant roots have higher nutrient concentrations, colonization space, and root metabolic activity, such as the root C/N ratio, specific root surface area, turnover rate, and respiration rate (Eissenstat et al., 2000; Pregitzer et al., 2002; Guo et al., 2004; Wang et al., 2017b), and deeper roots are oriented toward the acquisition and transport of nutrients and water (Germon et al., 2020). The enrichment effects of root diameters and rooting depths were similar to our bacterial diversity results in which higher bacterial diversity was found in thinner and deeper roots (Figure 1). However, it should be noted that the enrichment effect of the deepest roots decreased. This is similar to our previous study on the deep-rooted plant *Robinia pseudoacacia* L (Zai et al., 2021). The changes can be explained by the fact that the root density and soil nutrients decreased rapidly.

The core microbiota generally provides broad ecological functions (Shade and Handelsman, 2012). Not surprisingly, the genus *Ensifer*, which can establish highly effective nitrogen-fixing symbiosis with soybean plants (Albareda et al., 2009), was widely enriched in all root classes based on the results of differential enrichment analysis. Our study further showed that root diameter and rooting depth together significantly influenced the selection of root-associated core microbial taxa (Supplementary Figure S7). Root diameter mainly affected the core bacterial group in the endosphere; however, rooting depth mainly affected the core group in the rhizoplane and rhizosphere. The differential effects of root diameter and rooting depth on conserved core taxa in the whole system are worthy of attention, which suggests that root diameter and rooting depth are tightly linked to the potential vital functions of the root-associated microbial community in soybean. In our study, the core taxa *Novosphingobium*, *Bradyrhizobium*, *Neorhizobium*, *Stenotrophomonas*, *Klebsiella*, and *Variovorax* were associated with specific root diameter or rooting depth. These taxa have been reported to be related to soybean secretion of soyasaponins, nodulation, and resistance to biotic and abiotic stresses (Molla et al., 2001; Chen et al., 2016; Egamberdieva et al., 2016; Bianca et al., 2018; Liu et al., 2018; Fujimatsu et al., 2020). Therefore, root diameter and rooting depth play important roles in affecting the relationships between core beneficial microbes and specific root classes.

### Changes in Bacterial Co-occurrence Patterns Across Root Diameters and Rooting Depths

In natural ecosystems, microorganisms live together within complex networks that are linked through positive (commensalism and mutualism) and negative (amensalism and competition) interactions (Deng et al., 2012). In our study, the bacterial subnetworks of thinner and deeper roots were more complex (Figure 6 and Table 1), indicating that thinner and deeper roots promoted interactive processes in the community, playing positive roles in microbial coexistence (Goldford et al., 2018). The networks of thinner and deeper roots became more robust with higher network stability (Supplementary Figure S10), supporting the ecological belief that complexity begets stability (Montesinos-Navarro et al., 2017; Landi et al., 2018). In addition, higher positive correlations emerged in the networks of thinner and deeper roots, indicating that they included more cooperative or syntrophic associations (Karimi et al., 2017). These roots may rely on the associations between bacterial taxa to find and absorb nutrients and water.

Large variations in the bacterial module composition (functional ecological cluster) among different root diameters or rooting depths (Supplementary Figure S12) indicated that there was potential functional heterogeneity of the bacterial community among the different root classes (Newman, 2006). The keystone taxa belonging to *Bacillus*, *Ramlibacter* and *Ensifer* within the thinner root networks (Root I, Root II and Root III) are obviously beneficial for nutrient uptake and root development in an oligotrophic environment (Calvo et al., 2019; Props et al., 2019). *Sphingomonas* in the thicker root network (Root IV) can influence the water status and transport in root tissues (Asaf et al., 2017). The keystone taxa associated with the different root diameters indicate that thin roots more efficiently obtain nutrients and thick roots are more likely to transport nutrients. In terms of the bacterial networks of different rooting depths, the keystone taxa belonging to *Actinoplanes* and *Arthrobacter* in the shallower root networks (Layer I and Layer II) are associated with root stress tolerance (Sharma et al., 2016; Safdarian et al., 2019; El-Tarabily et al., 2020). *Pseudomonas* and *Ensifer* in the deeper root networks (Layer III and Layer IV) have mainly been shown to be related to the development and nutrient uptake of the root system (Cherni et al., 2019; Gan et al., 2020; Merino-Martín et al., 2020). These results indicated that the keystone taxa in deeper roots were more inclined to promote the nutrient uptake of roots. Overall, the complex associations of root-associated bacterial communities among root diameters and rooting depths appeared to be nonrandom and function driven.

## Conclusion

In summary, root diameter and rooting depth jointly affected the root-associated (rhizosphere, rhizoplane, and endosphere) bacterial communities of soybean plants. The structure, enrichment process, and network interactions of the bacterial community varied along the root diameter and rooting depth gradients. The thinner roots and deeper roots were inclined to enrich more diverse bacteria due to a higher hierarchical enrichment effect and to increase interspecific interactions of the root-associated bacterial community. Our results not only confirmed the bacterial niche differentiation from the rhizosphere soil to the root rhizoplane to the endosphere but also showed that there is a tuned adaptation or selective effect of the bacterial community among root diameters or rooting depths. These findings expand our understanding of relationships between bacterial communities and root traits and the complex host–microbe interactions in a root system.

## Data Availability Statement

The datasets presented in this study can be found in online repositories. The names of the repository/repositories and accession number(s) can be found in the article/Supplementary Material.

## Author Contributions

WC conceived and designed the study. GL helped with the experiment design. XZ, JS, YL, and DL helped with the collection of the samples. WL analyzed the data and wrote the manuscript. WC and GW helped with the improvement of the manuscript. All authors contributed to the article and approved the submitted version.

## Funding

This study was supported by the National Natural Science Foundation of China (31870476 and 41830755).

## Conflict of Interest

The authors declare that the research was conducted in the absence of any commercial or financial relationships that could be construed as a potential conflict of interest.

## Publisher’s Note

All claims expressed in this article are solely those of the authors and do not necessarily represent those of their affiliated organizations, or those of the publisher, the editors and the reviewers. Any product that may be evaluated in this article, or claim that may be made by its manufacturer, is not guaranteed or endorsed by the publisher.
