# Supplementary material for: Coupling Root Diameter With Rooting Depth to Reveal the Heterogeneous Assembly of Root-Associated Bacterial Communities in Soybean
Source: Front Microbiol. 2021 Dec 3;12:783563. doi: 10.3389/fmicb.2021.783563 (PMC8678505; doi:10.3389/fmicb.2021.783563)
Supplement: Supplementary file 1 [file Data_Sheet_1.PDF]

Supplementary information

The supplementary information includes six tables and eleven figures.

Summary

Table S1 Soil physical and chemical properties in different bulk soil layers (n=3). Values are mean ± standard deviation.

Table S2 General features of the high-throughout sequencing results of root diameters and rooting depths.

Table S3 Effects of rhizocompartment, root diameter and rooting depth on the bacterial community based on PERMANOVA.

Table S4 Pairwise comparisons of the bacterial community between root diameters or rooting depths in three rhizocompartments based on PERMANOVA.

Table S5 Dominant phyla with significant difference among root diameters or rooting depths (n=12). Values represent average relative abundances (%) (± standard deviation).

Table S6 Dominant genera with significant difference among root diameters or rooting depths (n=12). Values represent average relative abundances (%) (± standard deviation).

Fig. S1 Constrained analysis of principal coordinates (CAP) based on Bray-Curtis distances showed the root-associated bacterial communities are clearly separable among root diameters or rooting depths. (a) The variation in the root-associated bacterial community explained by root diameter when controlling for rhizocompartment and rooting depth; (b) The variation in the root-associated bacterial community explained by rooting depth when controlling for rhizocompartment and root diameter. Variance values were examined via PERMANOVA test, which are shown in each plot.

Fig. S2 Proportion (relative abundance) of enriched bacteria at genus level in each rhizocompartment among root diameters compared with bulk soil.

Fig. S3 Relative abundance of enriched bacteria at genus level in each rhizocompartment among rooting depths compared with bulk soil.

Fig. S4 Volcano diagrams of three rhizocompartments of different root diameters (a) and rooting depths (b). Enrichment (blue nodes) and depletion (red nodes) of OTUs for each rhizocompartment compared with bulk soil controls as determined by differential abundance analysis. Venn diagrams of the enriched and depleted OTUs of the microbiota colonized in rhizocompartments of shared among root diameters (c) or rooting depths (d).

Fig. S5 Relative abundance of specifically enriched bacteria at genus level in each rhizocompartment among root diameters compared with bulk soil.

Fig. S6 Relative abundance of specifically enriched bacteria at genus level in each rhizocompartment among rooting depths compared with bulk soil.

Fig. S7 Ratio and taxonomic distributions for the core taxa present in all samples. The proportion of OTU numbers (green line) and the relative abundance (blue line) of the core OTUs among root diameters (b) or rooting depths (c). Box plots showing the difference of relative abundance of the core OTUs among root diameters (b) or rooting depths (c).The boxes that do not share a letter are significantly different ($P < 0.05$; tested by multiple comparison with Kruskal-Wallis).

Fig. S8 Canonical discriminant analysis (CDA) plot comparing rhizocompartments of root diameters (a, b and c) and rooting depths (d, e and f) against bacterial taxa loadings, based on the

predominant (top 15 abundance) bacterial taxa at genus level. Arrows represent the degree of correlation between each taxon and each root class as a measure of the predictive discrimination of each root class. Circles represent the canonical group means and 85% confidence interval for each root class.

Fig. S9 Co-occurrence networks of the whole root and bulk soil. A connection stands for a strong (Spearman's $\rho > 0.6$) and significant ($P < 0.01$) correlation. The co-occurring networks are colored by module. The size of each node is proportional to the number of connections (i.e. degree). Positive links (PL), negative links (NL), the ratio of negative links to positive links (N/P), modularity (MD), average clustering coefficient (ACC), average path length (APL), average degree (AD).

Fig. S10 The robustness of the bacterial network of each root diameter (a) or rooting depth (b).

Fig. S11 A operational taxonomic unit (OTU) network map showing the composition and correlation of the OTUs among root diameters and rooting depths. OTUs in the left column were unique to one root diameter or rooting depth, while those in the right belonged to multiple root diameters or rooting depths. In total, 71 and 73 OTUs were found across all root diameters or rooting depths, accounting for more than 64.3% and 63.9%, respectively, of all the sequences in the experiment.

Fig. S12 Bacterial composition of modules in the network of each root diameter (a) or rooting depth (b) at phylum level.

Table S1 Soil physical and chemical properties in different bulk soil layers (n=3). Values are mean ± standard deviation.

	Soil Layer I (0-5cm)	Soil Layer II (5-10cm)	Soil Layer III (10-15cm)	Soil Layer IV (15-20cm)
TP (g/kg)	1.34 ± 0.11	1.32 ± 0.09	1.34 ± 0.08	1.34 ± 0.13
TK (g/kg)	16.69 ± 0.03	16.77 ± 0.02	16.52 ± 0.24	16.74 ± 0.05
TN (g/kg)	1.05 ± 0.05	0.91 ± 0.07	1.02 ± 0.11	0.97 ± 0.10
TOC (g/kg)	8.70 ± 0.38	8.03 ± 0.19	9.49 ± 1.49	8.34 ± 0.55
C/N	8.26 ± 0.08	8.93 ± 0.57	9.22 ± 0.6	8.67 ± 0.49
N/P	0.80 ± 0.10	0.70 ± 0.10	0.77 ± 0.11	0.75 ± 0.13
pH	8.49 ± 0.01	8.45 ± 0.01	8.43 ± 0.05	8.47 ± 0.05
SWC (%)	12.21 ± 0.21	12.12 ± 0.31	12.52 ± 0.13	12.45 ± 0.23
AK (mg/kg)	228.27 ± 25.97	190.2 ± 28.84	182.80 ± 37.67	172.27 ± 33.03
AP (mg/kg)	14.43 ± 1.40	17.62 ± 0.81	18.13 ± 2.74	14.99 ± 1.73
NO_3^--N (mg/kg)	0.70 ± 0.21	0.96 ± 0.37	1.17 ± 0.34	1.09 ± 0.62
NH_4^+-N (mg/kg)	3.81 ± 1.69	7.01 ± 2.29	5.92 ± 1.34	4.62 ± 2.44

Note: TP: total phosphate, TN: total nitrogen, TP: total potassium, TOC: total organic carbon, C/N: ratio of total carbon to total nitrogen, N/P: ratio of total nitrogen to total phosphorus, SWC: soil water content, AK: available potassium, AP: available phosphorus, NO_3^--N: nitrate nitrogen, NH_4^+-N: ammonium nitrogen.

.

Table S2 General features of the high-throughout sequencing results of root diameters and rooting depths.

Sample name	Rhizocompartment	Raw reads	Clean reads	Number of OTU(97%)	Numbere of genus	Number of family	Number of order	Number of class	Number of phylum	Diversity		Richness			Evenness	Goods coverage
										Shannon	Inversimpson	Observed_species	Chao1	ACE	Pielou evenness	
Root I	Endosphere	1003480	967570	2799	367	168	96	47	33	4.126±0.634	4.556±1.244	783.083±165.400	1134.363±217.691	1180.94±228.336	0.619±0.078	0.991±0.002
Root I	Rhizoplane	985051	919702	4462	512	218	118	54	43	6.54±1.627	38.281±34.532	1462.000±452.265	1905.571±504.874	1963.521±489.845	0.897±0.188	0.987±0.003
Root I	Rhizosphere	1007328	954801	5191	489	211	113	50	40	7.781±0.995	63.956±71.115	2171.917±241.153	2855.228±281.949	2875.167±235.694	1.012±0.119	0.981±0.002
Root II	Endosphere	1010651	966570	2765	382	180	100	47	32	4.269±0.664	4.565±1.574	785.000±133.859	1137.851±179.811	1184.129±179.414	0.640±0.085	0.991±0.001
Root II	Rhizoplane	1020365	957679	4342	532	226	117	53	43	6.386±1.700	36.223±31.842	1324.500±376.628	1618.225±438.084	1685.83±458.737	0.887±0.212	0.99±0.004
Root II	Rhizosphere	946835	895316	4752	475	207	109	48	36	6.873±1.365	30.021±26.442	1764.417±376.126	2324.944±435.657	2378.06±419.519	0.918±0.161	0.984±0.003
Root III	Endosphere	997308	962185	2453	368	171	96	44	28	3.524±0.528	3.284±0.636	636.667±123.514	928.950±207.3796	968.122±207.229	0.546±0.069	0.993±0.002
Root III	Rhizoplane	994462	931838	3890	490	217	115	50	39	5.856±1.600	21.732±18.799	1134.333±393.622	1454.093±425.715	1507.086±433.628	0.835±0.21	0.99±0.0.003
Root III	Rhizosphere	990274	920892	4772	481	206	110	49	36	6.904±1.467	32.038±28.797	1781.583±422.075	2320.166±482.546	2372.528±477.250	0.921±0.171	0.984±0.003
Root IV	Endosphere	991926	962020	2317	366	169	92	45	31	3.383±0.916	3.604±1.507	555.500±164.414	811.578±209.877	843.149±213.146	0.534±0.122	0.994±0.001
Root IV	Rhizoplane	966260	911876	3420	468	199	111	51	38	5.296±1.113	15.164±14.931	943.667±256.231	1261.294±287.468	1311.489±305.561	0.774±0.14	0.991±0.002
Root IV	Rhizosphere	992753	938129	4482	481	207	110	50	38	6.294±1.509	30.698±40.062	1525.917±514.892	2046.378±634.066	2089.906±615.41	0.859±0.171	0.986±0.004
Layer I	Endosphere	1007915	967618	2496	364	167	91	41	27	3.845±0.725	3.762±1.166	686.833±148.421	982.800±230.646	1017.972±233.011	0.588±0.098	0.993±0.002
Layer I	Rhizoplane	1056672	985544	3619	483	213	113	53	40	5.352±1.458	17.98±15.218	995.083±289.559	1341.954±429.158	1393.512±435.964	0.779±0.209	0.991±0.004
Layer I	Rhizosphere	1009660	958761	4520	458	204	111	50	41	7.161±1.263	49.258±55.693	1821.917±297.149	2413.094±325.183	2459.549±319.216	0.953±0.155	0.983±0.002
Layer II	Endosphere	1003664	962557	2685	377	168	95	45	32	4.099±0.732	4.603±1.674	753.667±151.606	1078.350±207.121	1125.626±202.714	0.618±0.094	0.992±0.001
Layer II	Rhizoplane	1019882	956055	3732	484	217	116	52	40	5.582±1.732	24.892±27.367	1025.000±464.806	1351.898±490.059	1394.819±494.183	0.805±0.201	0.991±0.003

Layer II	Rhizosphere	993188	931146	4701	482	199	106	48	37	6.470±1.773	32.532±40.815	1613.833±560.348	2163.574±648.483	2213.471±635.131	0.875±0.201	0.985±0.004
Layer III	Endosphere	991762	962273	2641	366	176	101	46	33	3.735±0.923	3.931±1.448	675.500±231.051	1003.814±313.035	1042.397±332.788	0.573±0.113	0.992±0.002
Layer III	Rhizoplane	965410	903217	4217	499	216	118	52	41	6.473±1.158	30.481±29.313	1374.000±300.724	1739.843±367.221	1792.616±365.959	0.896±0.139	0.988±0.003
Layer III	Rhizosphere	984765	926066	4855	477	212	114	50	37	6.820±1.482	37.099±51.450	1787.667±513.257	2361.531±622.963	2391.407±582.550	0.910±0.166	0.984±0.004
Layer IV	Endosphere	1000024	965897	2535	369	170	96	46	32	3.623±0.733	3.713±1.142	644.250±156.063	947.776±217.578	990.345±218.138	0.560±0.094	0.993±0.001
Layer IV	Rhizoplane	924174	876279	4470	513	220	118	52	41	6.671±1.591	38.047±33.433	1470.417±390.923	1805.488±441.486	1886.980±439.596	0.913±0.191	0.988±0.003
Layer IV	Rhizosphere	949577	893165	5076	497	216	111	51	37	7.401±1.022	37.824±38.803	2020.417±346.796	2608.517±505.173	2651.234±484.962	0.972±0.114	0.982±0.004
Bulk Soil	Bulk Soil	1022754	964325	5470	449	202	114	57	48	9.614±0.152	274.051±80.161	2830.667±137.428	3472.733±184.374	3422.340±178.006	1.210±0.015	0.980±0.001
Bulk Soil	Bulk Soil I	256344	240458	4528	390	183	108	51	39	9.559±0.092	199.73±86.704	2895.000±126.004	3590.766±134.160	3525.156±140.706	1.199±0.013	0.979±0.001
Bulk Soil	Bulk Soil II	263141	243130	4356	360	174	104	52	43	9.619±0.091	282.93±34.375	2815.667±66.561	3446.462±132.465	3390.913±71.293	1.211±0.009	0.980±0.001
Bulk Soil	Bulk Soil III	253254	240392	4362	358	176	99	49	39	9.637±0.19	297.752±93.883	2792.333±116.543	3364.505±166.799	3338.006±164.996	1.215±0.018	0.981±0.002
Bulk Soil	Bulk Soil IV	250015	240345	4485	399	185	108	53	44	9.641±0.260	315.790±77.261	2819.667±247.092	3489.199±289.290	3435.286±306.449	1.214±0.02	0.979±0.002

Note: Values of diversity, richness, evenness, and goods_coverage of every root diameter or rooting depth in each rhizocompartment represent average value (± standard error) of twelve replicates.

Table S3 Effects of rhizocompartment, root diameter and rooting depth on the bacterial community based on PERMANOVA.

Group	Df	SumsOfSqs	MeanSqs	F.Model	R^2	Pr (>F).
			**Whole data**			
Rhizocompartment	2	17.536625	8.7683126	68.582051	0.4624209	0.001
Rooting depth	3	1.805176	0.6017253	4.7064423	0.0476004	0.001
Root diameter	3	1.3217711	0.4405904	3.4461124	0.0348536	0.001
Residuals	135	17.259942	0.1278514		0.4551251	
Total	143	37.923514			1	
			**Endosphere**			
Rooting depth	3	0.1920519	0.0640173	1.4881833	0.0863201	0.115
Root diameter	3	0.2691284	0.0897095	2.0854387	0.1209631	0.021
Residuals	41	1.7637002	0.0430171		0.7927168	
			**Rhizoplane**			
Rooting depth	3	1.619128	0.5397093	2.945566	0.1635948	0.001
Root diameter	3	0.765719	0.2552397	1.3930188	0.0773674	0.073
Residuals	41	7.5123362	0.1832277		0.7590378	
			**Rhizosphere**			
Rooting depth	3	1.0935097	0.3645032	2.3399061	0.1323089	0.001
Root diameter	3	0.7844636	0.2614879	1.6786053	0.0949159	0.018
Residuals	41	6.3868517	0.1557769		0.7727752	

Table S4 Pairwise comparisons of the bacterial community between root diameters or rooting depths in three rhizocompartments based on PERMANOVA.

Pairs	SumsOfSqs	MeanSqs	F.Model	R^2	Pr(>F)	sig	Rhizocompartment
				**Whole data**			
Endosphere VS Rhizoplane	12.29597323	12.29597323	95.34857394	0.503561088	0.000999001	***	
Endosphere VS Rhizosphere	12.92583406	12.92583406	115.8305534	0.552019482	0.000999001	***	
Rhizoplane VS Rhizosphere	1.083130539	1.083130539	5.605892807	0.056280734	0.000999001	***	
Layer I VS Layer II	0.650244925	0.650244925	2.419371411	0.033407794	0.041958042	*	
Layer I VS Layer III	0.570062028	0.570062028	2.179738206	0.030198755	0.085914086		
Layer I VS Layer IV	0.845017824	0.845017824	3.325361087	0.045350763	0.00999001	**	
Layer II VS Layer III	0.509271438	0.509271438	1.944799917	0.027031834	0.061938062		
Layer II VS Layer IV	0.818257193	0.818257193	3.215806631	0.0439223	0.018981019	*	
Layer III VS Layer IV	0.217498548	0.217498548	0.879812211	0.012412733	0.3996004		
Root I VS Root II	0.296012523	0.296012523	1.208292838	0.016968429	0.248751249		
Root I VS Root III	0.302870251	0.302870251	1.185041904	0.016647344	0.242757243		
Root I VS Root IV	0.752812075	0.752812075	2.831345153	0.038875365	0.024975025	*	
Root II VS Root III	0.149356128	0.149356128	0.581158837	0.008233909	0.7002997		
Root II VS Root IV	0.708984807	0.708984807	2.652350247	0.036507425	0.028971029	*	
Root III VS Root IV	0.433506396	0.433506396	1.559948168	0.02179918	0.144855145		
				**Root diameter**			
Root I VS Root II	0.236774	0.236774	1.59288	0.067515	0.115884116		Rhizosphere
Root I VS Root III	0.193637	0.193637	1.288345	0.055321	0.20979021		Rhizosphere
Root I VS Root IV	0.438569	0.438569	2.493684	0.101809	0.00999001	**	Rhizosphere
Root II VS Root III	0.100373	0.100373	0.611494	0.027044	0.798201798		Rhizosphere
Root II VS Root IV	0.333181	0.333181	1.756197	0.073926	0.078921079		Rhizosphere
Root III VS Root IV	0.266393	0.266393	1.392021	0.059508	0.174825175		Rhizosphere
Root I VS Root II	0.166888	0.166888	0.872489	0.038146	0.589410589		Rhizoplane
Root I VS Root III	0.182164	0.182164	0.89085	0.038917	0.543456543		Rhizoplane
Root I VS Root IV	0.403302	0.403302	1.9165	0.080133	0.04995005	*	Rhizoplane
Root II VS Root III	0.092268	0.092268	0.450901	0.020084	0.985014985		Rhizoplane
Root II VS Root IV	0.417168	0.417168	1.981014	0.082608	0.017982018	*	Rhizoplane
Root III VS Root IV	0.269648	0.269648	1.204924	0.051925	0.260739261		Rhizoplane
Root I VS Root II	0.056037	0.056037	1.242026	0.053439	0.226773227		Endosphere
Root I VS Root III	0.102679	0.102679	2.586377	0.105196	0.056943057		Endosphere

Root I VS Root IV	0.128651	0.128651	2.501463	0.102094	0.046953047	*	Endosphere
Root II VS Root III	0.08125	0.08125	2.168553	0.089726	0.092907093		Endosphere
Root II VS Root IV	0.131353	0.131353	2.669898	0.108225	0.028971029	*	Endosphere
Root III VS Root IV	0.038286	0.038286	0.874506	0.038231	0.394605395		Endosphere
				**Rooting depth**			
Layer I VS Layer II	0.400557	0.400557	2.321927	0.095466	0.023976024	*	Rhizosphere
Layer I VS Layer III	0.265919	0.265919	1.579069	0.066969	0.12987013		Rhizosphere
Layer I VS Layer IV	0.46809	0.46809	3.503495	0.137373	0.000999001	***	Rhizosphere
Layer II VS Layer III	0.274751	0.274751	1.428298	0.060965	0.180819181		Rhizosphere
Layer II VS Layer IV	0.559765	0.559765	3.552557	0.139029	0.001998002	**	Rhizosphere
Layer III VS Layer IV	0.217938	0.217938	1.42018	0.060639	0.162837163		Rhizosphere
Layer I VS Layer II	0.537158	0.537158	2.476735	0.101187	0.002997003	**	Rhizoplane
Layer I VS Layer III	0.54248	0.54248	2.938905	0.117844	0.006993007	**	Rhizoplane
Layer I VS Layer IV	0.666933	0.666933	3.393782	0.133646	0.002997003	**	Rhizoplane
Layer II VS Layer III	0.625198	0.625198	3.477979	0.136509	0.003996004	**	Rhizoplane
Layer II VS Layer IV	0.715562	0.715562	3.732921	0.145064	0.003996004	**	Rhizoplane
Layer III VS Layer IV	0.150925	0.150925	0.946869	0.041264	0.498501499		Rhizoplane
Layer I VS Layer II	0.069874	0.069874	1.447347	0.061728	0.20979021		Endosphere
Layer I VS Layer III	0.117264	0.117264	2.907009	0.116714	0.024975025	*	Endosphere
Layer I VS Layer IV	0.073284	0.073284	1.695658	0.07156	0.124875125		Endosphere
Layer II VS Layer III	0.050297	0.050297	1.022654	0.044419	0.387612388		Endosphere
Layer II VS Layer IV	0.040901	0.040901	0.785605	0.034478	0.506493506		Endosphere
Layer III VS Layer IV	0.032484	0.032484	0.736195	0.03238	0.554445554		Endosphere

Note: *, $P < 0.05$; **, $P < 0.01$; ***, $P < 0.001$.

Table S5 Dominant phyla with significant difference among root diameters or rooting depths (n=12). Values represent average relative abundances (%) (± standard deviation).

Phylum	Root I	Root II	Root III	Root IV	Layer I	Layer II	Layer III	Layer IV
				**Rhizosphere**				
Deltaproteobacteria	2.73±1.17a	1.92±1.00ab	1.86±0.88ab	1.37±0.91b	2.20±1.20	1.61±1.22	1.88±1.04	2.19±0.87
Oxyphotobacteria	0.76±0.82	0.59±0.49	0.80±0.76	0.59±0.62	0.70±0.68ab	0.49±0.71b	0.64±0.55ab	0.91±0.73a
Bacteroidetes	7.06±3.48a	3.64±2.25b	5.24±3.61ab	3.51±2.47b	6.67±4.48	5.25±2.88	3.34±2.06	4.19±2.46
Acidobacteria	4.23±1.73a	2.82±1.53ab	2.81±1.52ab	1.70±1.05b	2.66±1.72	2.47±2.07	3.12±1.95	3.32±0.83
Chloroflexi	3.79±1.37a	2.49±1.19ab	2.49±1.33ab	1.47±0.97b	2.06±0.95	2.20±1.75	2.77±1.6	3.21±1.23
Verrucomicrobia	1.76±1.21a	1.01±0.7ab	0.85±0.46ab	0.57±0.42b	1.30±0.96	0.94±1.10	0.98±0.84	0.97±0.49
Gemmatimonadetes	0.44±0.21a	0.18±0.14b	0.17±0.10b	0.17±0.12b	0.34±0.24	0.2±0.19	0.21±0.16	0.20±0.10
				**Rhizoplane**				
Alphaproteobacteria	34.02±14.13	37.39±18.76	35.03±17.14	38.53±19.85	40.99±19.98a	20.51±12.55b	44.01±15.17a	39.46±9.75a
Deltaproteobacteria	1.51±0.11a	2.21±2.22a	0.98±0.56ab	0.53±0.39b	0.72±0.46	1.35±2.19	1.37±0.69	1.79±1.34
Gammaproteobacteria	41.01±23.65	38.53±17.87	46.68±21.82	50.44±24.23	44.62±18.82ab	64.77±23.74a	34.34±11.87b	32.92±16.5b
Oxyphotobacteria	0.81±0.80ab	2.84±3.45a	1.22±1.35ab	0.45±0.87b	0.66±0.91ab	0.82±1.64b	1.52±1.47a	2.33±3.34a
Actinobacteria	6.63±4.98	7.22±3.38	6.93±4.17	5.21±4.22	5.68±4.63ab	4.32±3.58b	7.53±3.54a	8.46±3.99a
Bacteroidetes	7.67±10.14a	2.77±1.76ab	2.70±1.98ab	1.65±1.01b	2.53±1.90	2.49±2.18	3.88±3.20	5.90±10.38
Acidobacteria	1.95±1.43a	1.50±0.96a	1.28±0.85a	0.54±0.43b	0.65±0.45c	0.98±0.97bc	1.67±1.01ab	1.95±1.29a
Chloroflexi	2.21±1.65a	2.24±2.00a	1.53±1.26a	0.47±0.37b	0.99±1.17	1.47±2.02	1.61±1.01	2.39±1.76
Planctomycetes	0.84±0.56	1.29±1.41	0.69±0.47	0.38±0.31	0.45±0.29b	0.77±1.34b	0.81±0.4ab	1.18±0.86a
Gemmatimonadetes	0.14±0.09a	0.10±0.06a	0.07±0.05ab	0.05±0.04b	0.08±0.06	0.07±0.08	0.10±0.05	0.12±0.08
				**Endosphere**				
Deltaproteobacteria	0.49±0.23a	0.58±0.23a	0.40±0.20a	0.23±0.14b	0.49±0.23	0.58±0.23	0.40±0.20	0.23±0.14
Gammaproteobacteria	5.56±3.27	4.72±2.30	4.10±2.74	3.34±2.62	5.56±3.27ab	4.72±2.30a	4.10±2.74b	3.34±2.62b
Oxyphotobacteria	64.62±8.77b	65.38±10.44b	75.52±7.26a	75.29±11.58a	64.62±8.77	65.38±10.44	75.52±7.26	75.29±11.58
Actinobacteria	7.01±3.15ab	10.49±4.04a	4.96±2.49bc	3.82±3.48c	7.01±3.15	10.49±4.04	4.96±2.49	3.82±3.48
Bacteroidetes	1.62±1.51a	1.17±0.65ab	0.74±0.53bc	0.82±0.98c	1.62±1.51	1.17±0.65	0.74±0.53	0.82±0.98
Acidobacteria	0.30±0.14a	0.32±0.16a	0.23±0.13ab	0.15±0.10b	0.30±0.14	0.32±0.16	0.23±0.13	0.15±0.10
Chloroflexi	0.43±0.25a	0.45±0.19a	0.22±0.12b	0.13±0.09b	0.43±0.25	0.45±0.19	0.22±0.12	0.13±0.09
Planctomycetes	0.11±0.06ab	0.17±0.06a	0.09±0.05b	0.09±0.08b	0.11±0.06	0.17±0.06	0.09±0.05	0.09±0.08

Note: Different letters meant significant difference between root diameters or rooting depth at 0.05 level (multiple comparison with Kruskal–Wallis tests). RS, rhizosphere; RP, rhizoplane; RE, endosphere.

Table S6 Dominant genera with significant difference among root diameters or rooting depths (n=12). Values represent average relative abundances (%) (± standard deviation).

Genus	Root I	Root II	Root III	Root IV	Layer I	Layer II	Layer III	Layer IV
					**Rhizosphere**			
Klebsiella	4.80±8.18	2.54±5.72	6.32±12.46	6.65±10.7	3.31±4.4a	12.57±15.47a	3.80±5.79ab	0.63±0.86b
Chryseobacterium	2.05±2.85	0.8±1.04	2.43±3.83	1.30±1.58	2.24±3.68ab	2.53±2.97a	0.67±0.83b	1.14±1.59ab
					**Rhizoplane**			
Ensifer	12.64±14.17	10.57±19.35	8.42±7.62	11.71±13.82	8.9±19.87b	5.3±4.52b	16.41±15.65a	12.72±9.97a
Pseudomonas	12.08±11.77	9.98±9.28	14.33±19.24	13.89±12.84	15.25±12.6ab	18.1±14.17a	5.11±3.51b	11.83±17.27ab
Klebsiella	6.29±12.43	4.08±6.77	8.48±16.86	8.54±10.64	3.39±4.23b	17.53±18.69a	5.23±8.22b	1.24±1.74b
Stenotrophomonas	2.48±2.32	4.5±6.25	4.06±4.84	2.53±3.45	7.27±6.55a	2.32±1.48ab	2.79±3.8ab	1.19±0.67b
Neorhizobium	2.85±2.17b	2.68±1.26b	2.93±1.48ab	6.00±4.32a	5.16±4.02a	2.43±2.68b	4.2±2.01a	2.66±1.60b
Novosphingobium	2.13±1.19	3.56±2.23	3.59±3.01	1.88±1.65	1.78±1.03b	1.77±1.35b	3.49±1.86a	4.12±3.14a
Streptomyces	1.84±2.15	2.30±1.36	1.88±1.42	1.13±1.16	1.31±2.2b	1.14±0.99b	2.27±1.18a	2.43±1.41a
Variovorax	2.22±1.47	2.17±0.91	2.05±1.33	2.05±1.55	1.58±1.07b	1.42±0.97b	3.30±1.26a	2.19±1.06ab
					**Endosphere**			
Pseudomonas	0.30±0.61	0.03±0.03	0.10±0.24	0.31±0.59	0.36±0.57a	0.33±0.63b	0.03±0.07b	0.02±0.03b
Klebsiella	0.58±1.29	0.10±0.22	0.42±0.88	0.22±0.46	0.22±0.55ab	0.93±1.38a	0.09±0.23bc	0.08±0.22c
Pseudochrobactrum	0.06±0.06	0.05±0.05	0.02±0.03	0.03±0.04	0.08±0.07a	0.03±0.03b	0.02±0.03b	0.02±0.02b

Note: Different letters indicate significant difference between root diameters or rooting depth at 0.05 level (multiple comparison with Kruskal–Wallis tests). RS, rhizosphere; RP, rhizoplane; RE, endosphere.

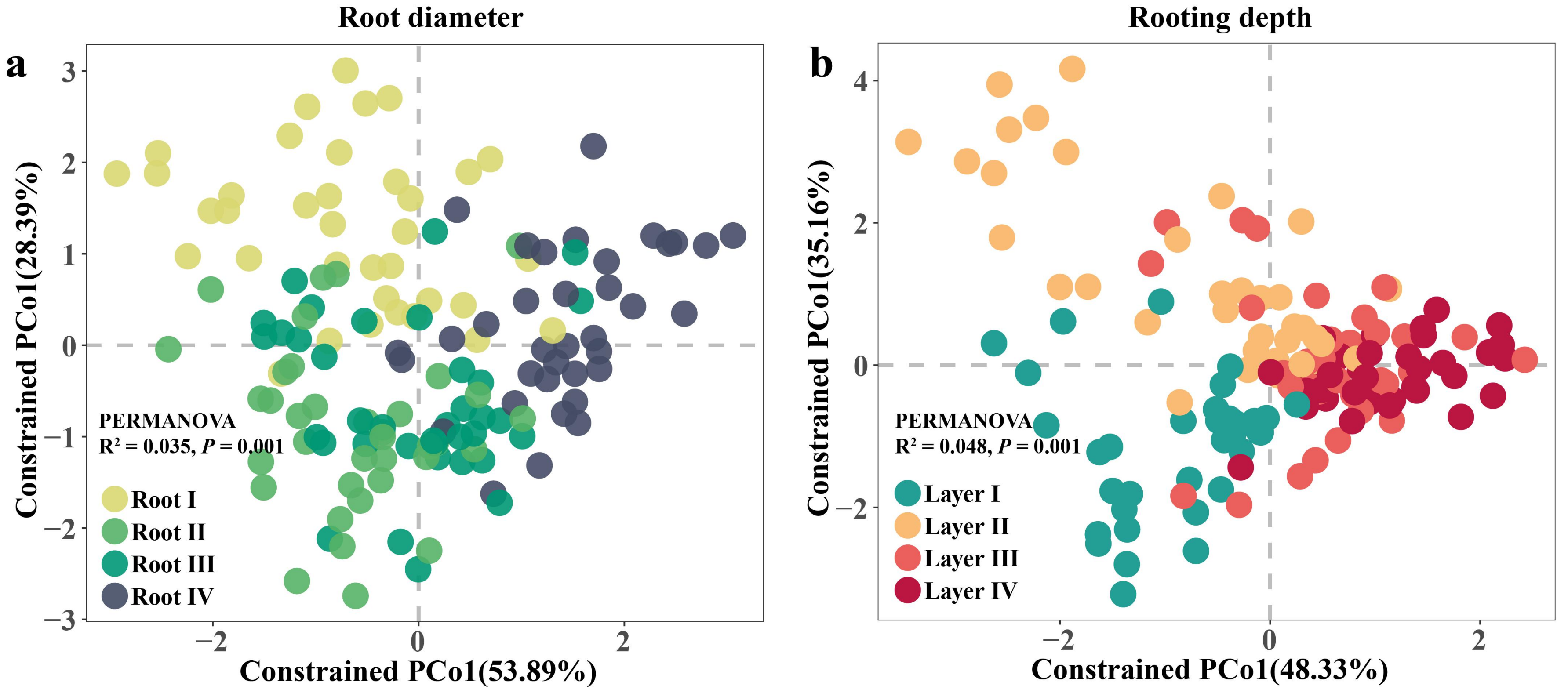

Fig. S1 Constrained analysis of principal coordinates (CAP) based on Bray-Curtis distances showed the root-associated bacterial communities are clearly separable among root diameters or rooting depths. (a) The variation in the root-associated bacterial community explained by root diameter when controlling for rhizocompartment and rooting depth; (b) The variation in the root-associated bacterial community explained by rooting depth when controlling for rhizocompartment and root diameter. Variance values were examined via PERMANOVA test, which are shown in each plot.

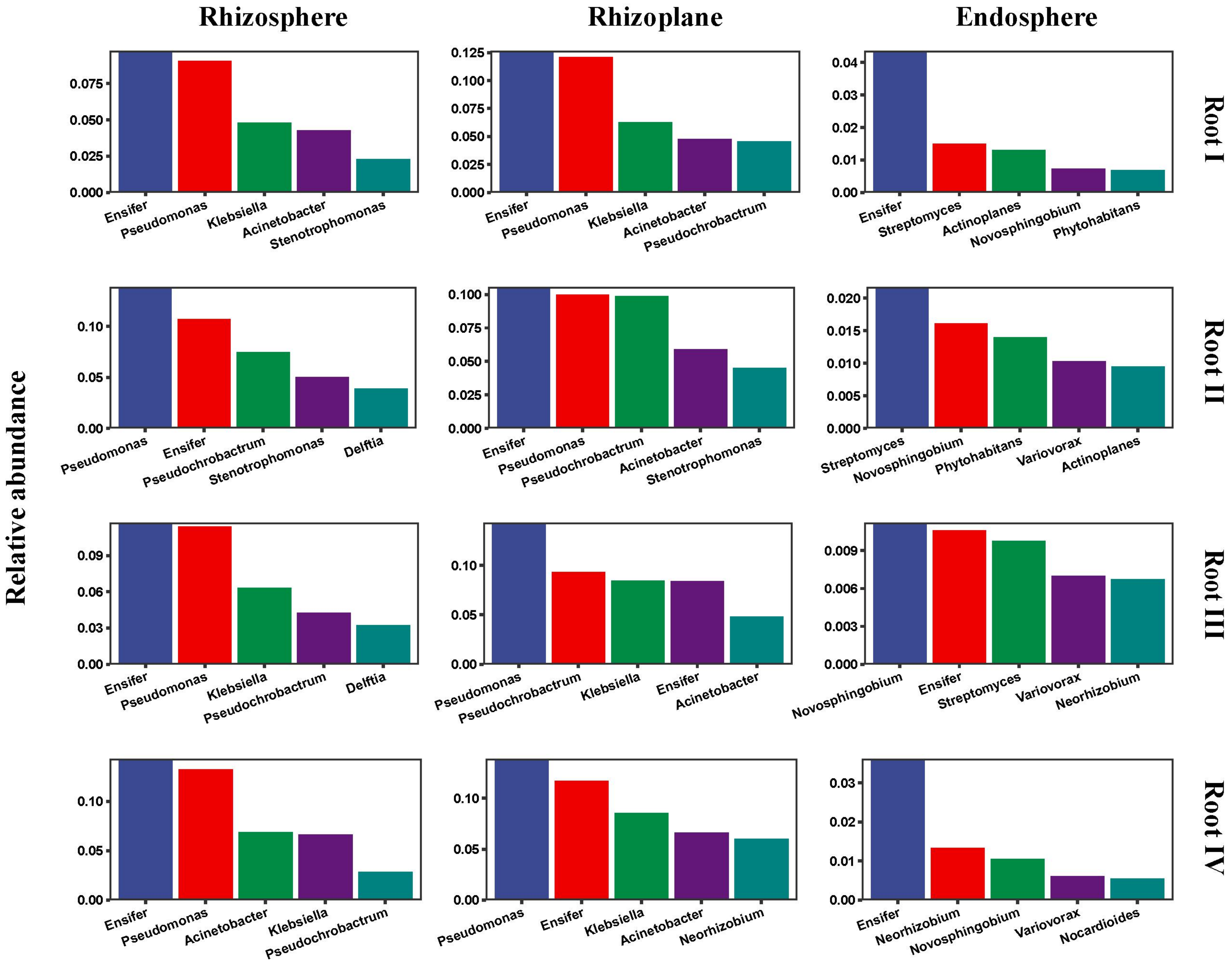

Fig. S2 Relative abundance of enriched bacteria at genus level in each rhizocompartment among root diameters compared with bulk soil.

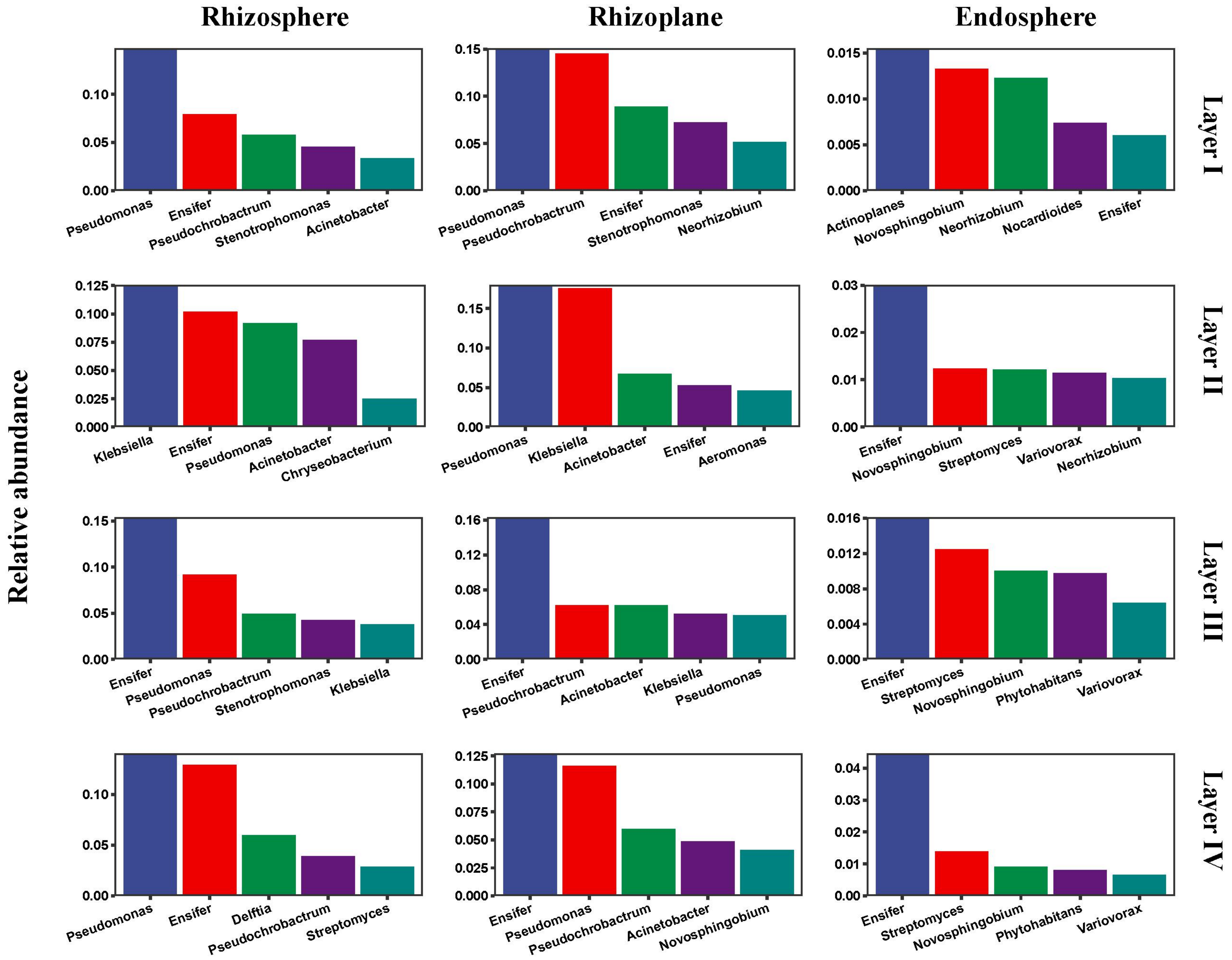

Fig. S3 Relative abundance of enriched bacteria at genus level in each rhizocompartment among rooting depths compared with bulk soil.

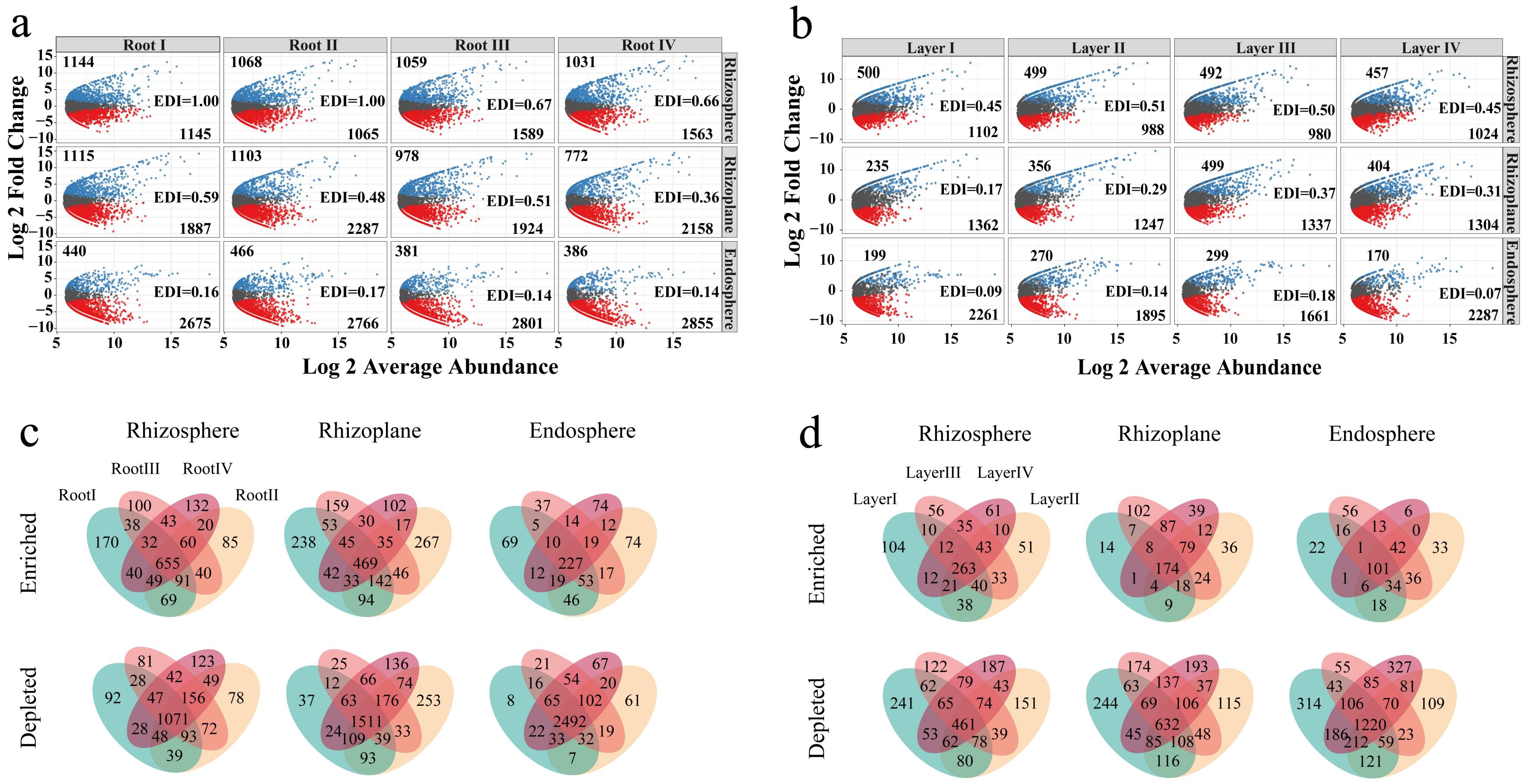

Fig. S4 Volcano diagrams of three rhizocompartments of different root diameters (a) and rooting depths (b). Enrichment (blue nodes) and depletion (red nodes) of OTUs for each rhizocompartment compared with bulk soil controls as determined by differential abundance analysis. Venn diagrams of the enriched and depleted OTUs of the microbiota colonized in rhizocompartments of shared among root diameters (c) or rooting depths (d).

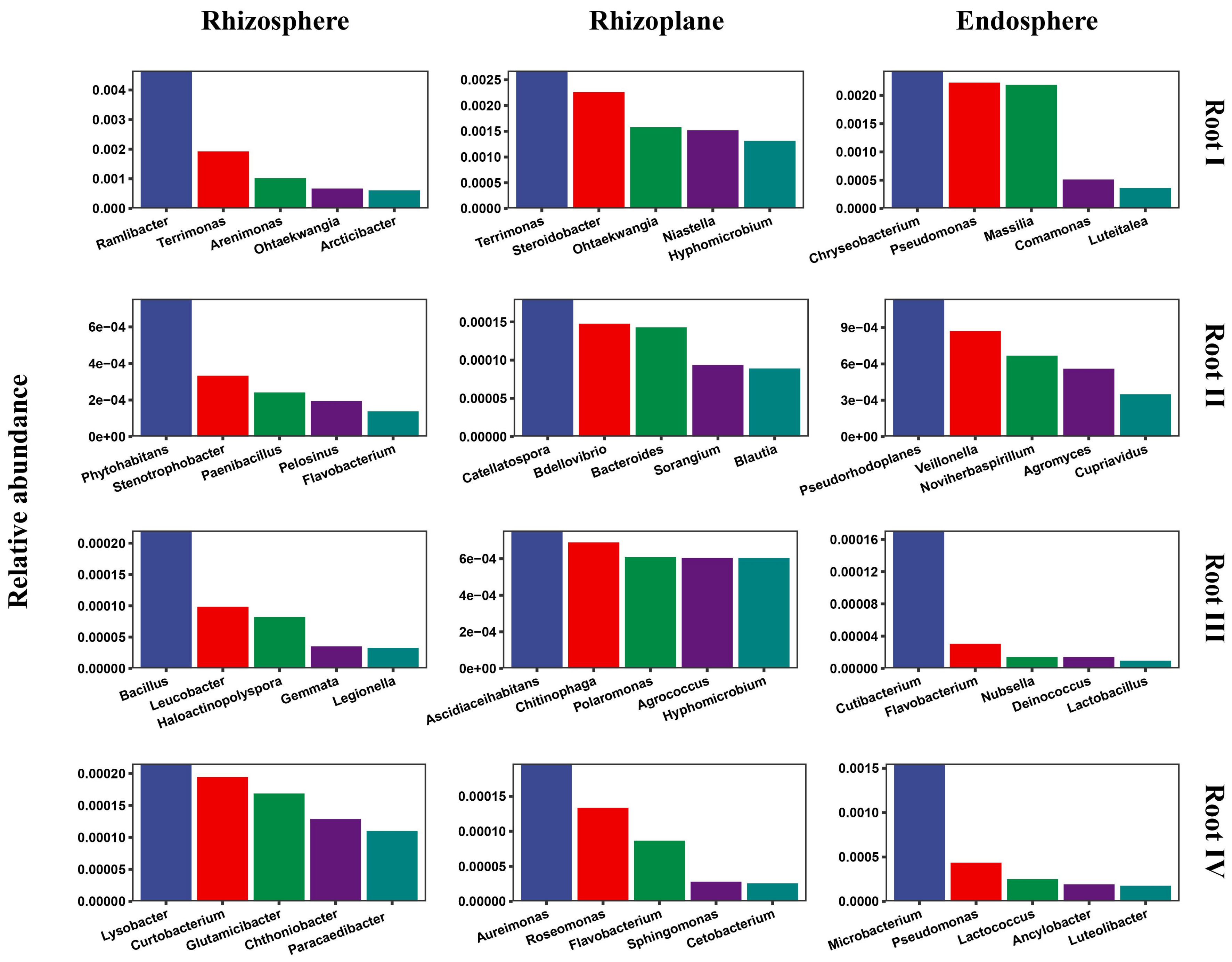

Fig. S5 Relative abundance of specifically enriched bacteria at genus level in each rhizocompartment among root diameters compared with bulk soil.

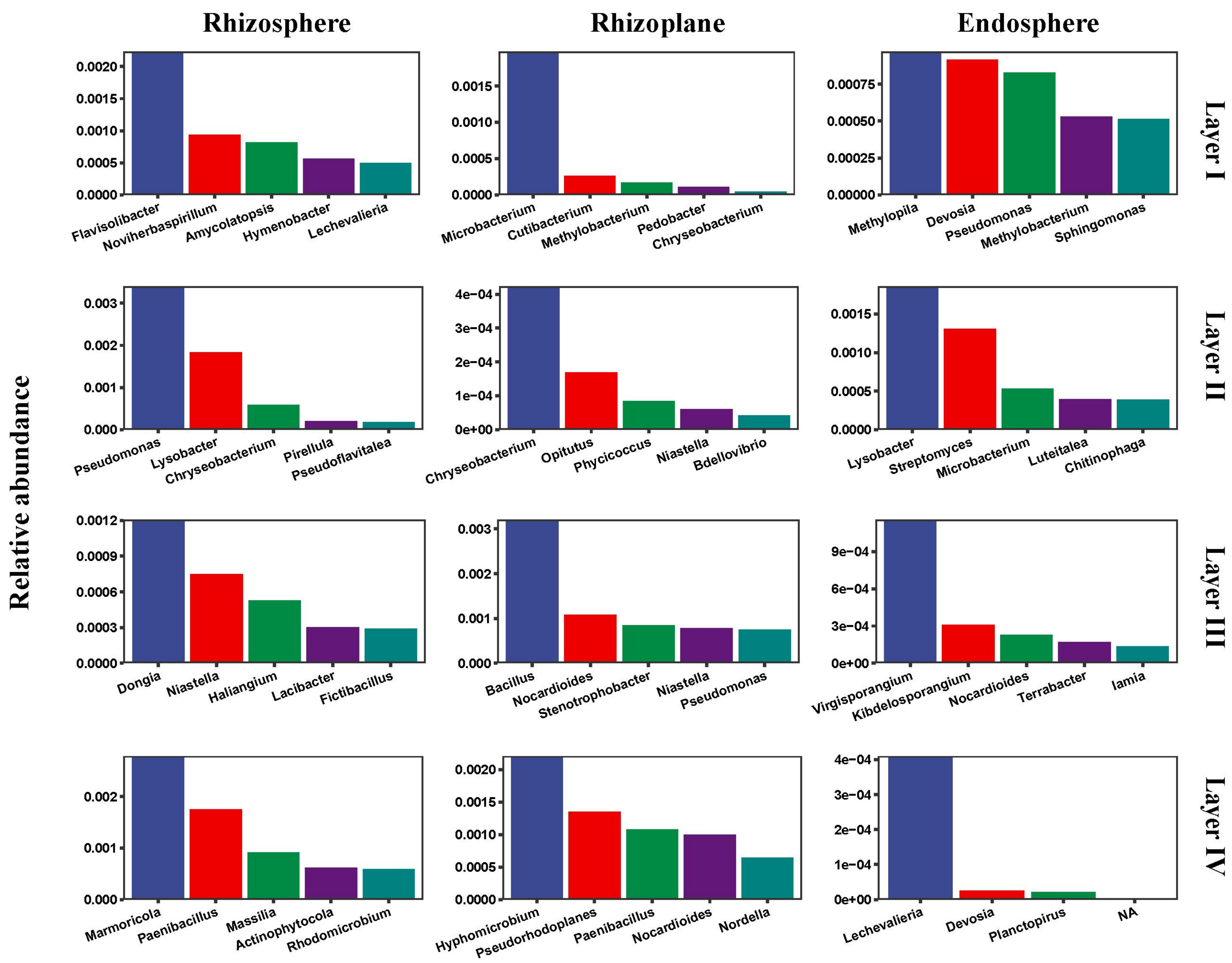

Fig. S6 Relative abundance of specifically enriched bacteria at genus level in each rhizocompartment among rooting depths compared with bulk soil.

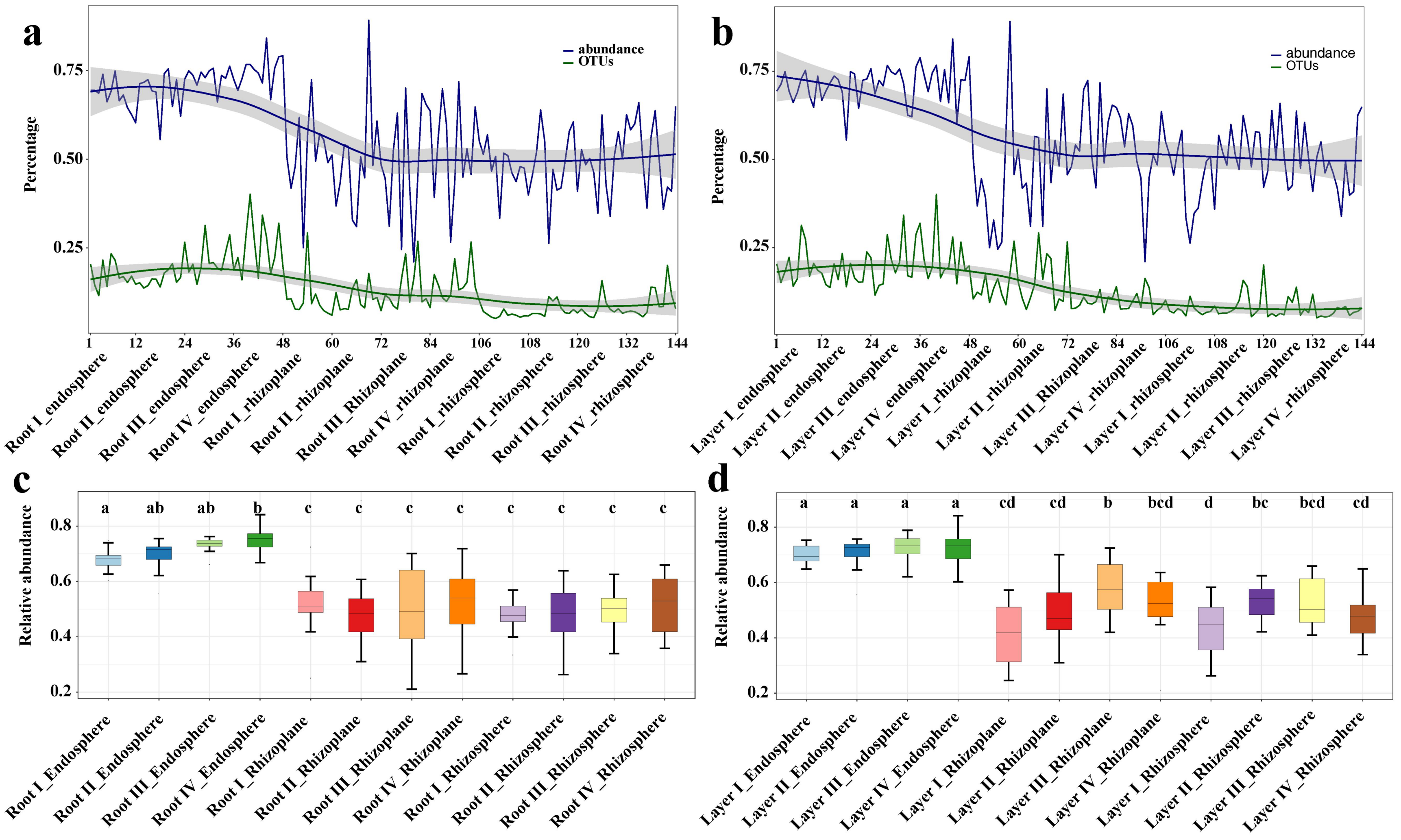

Fig. S7 Ratio and taxonomic distributions for the core taxa present in all samples. The proportion of OTU numbers (green line) and the relative abundance (blue line) of the core OTUs among different root diameters (b) and rooting depths (c). Box plots showing the difference of relative abundance of the core OTUs among root diameters (b) or rooting depths (c).The boxes that do not share a letter are significantly different ($P < 0.05$; tested by multiple comparison with Kruskal-Wallis).

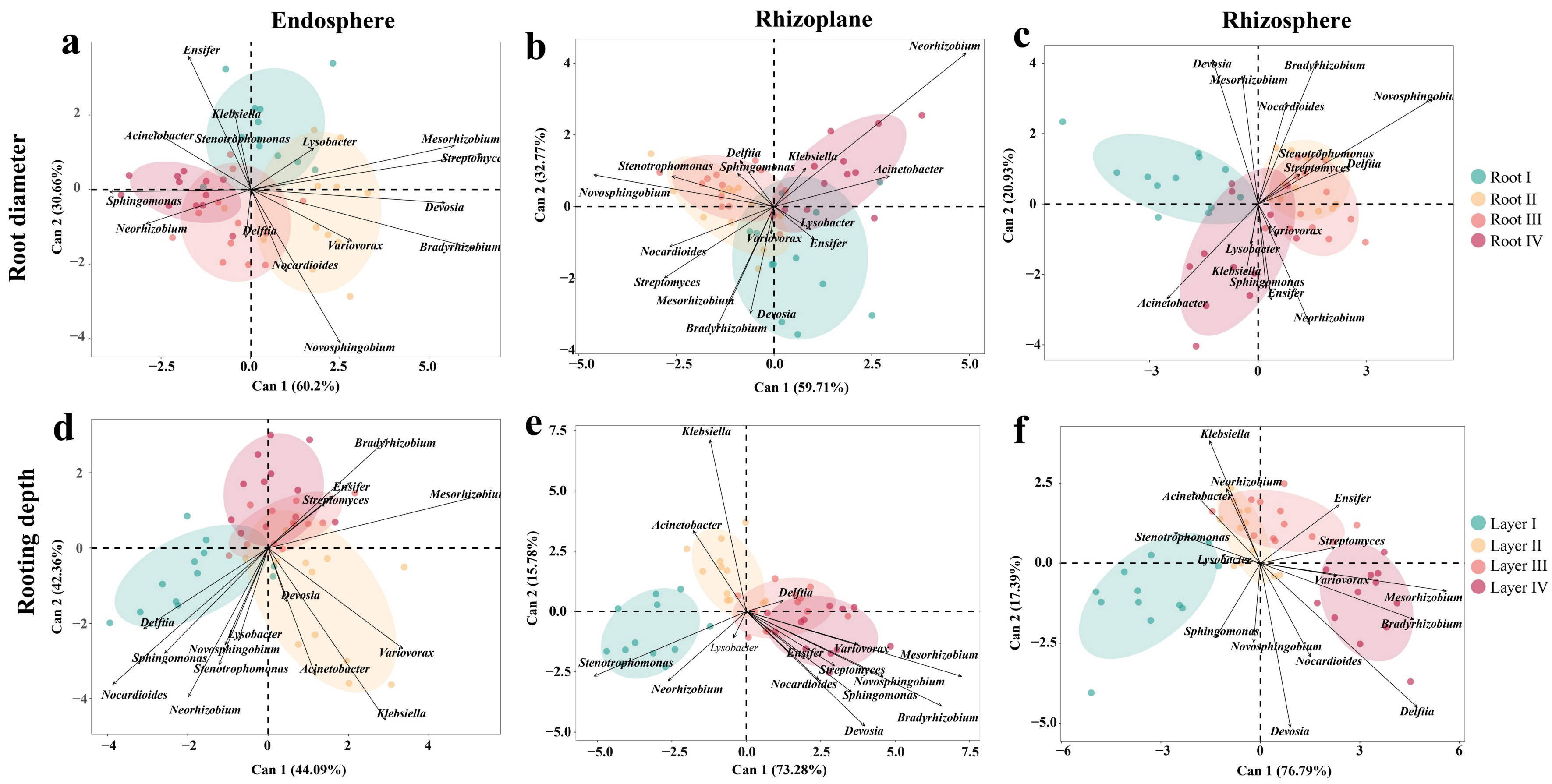

Fig. S8 Canonical discriminant analysis (CDA) plot comparing rhizocompartments of root diameters (a, b and c) and rooting depths (d, e and f) against bacterial taxa loadings, based on the predominant (top 15 abundance) bacterial taxa at genus levels. Arrows represent the degree of correlation between each taxon and each root class as a measure of the predictive discrimination of each root class. Circles represent the canonical group means and 85% confidence interval for each root class.

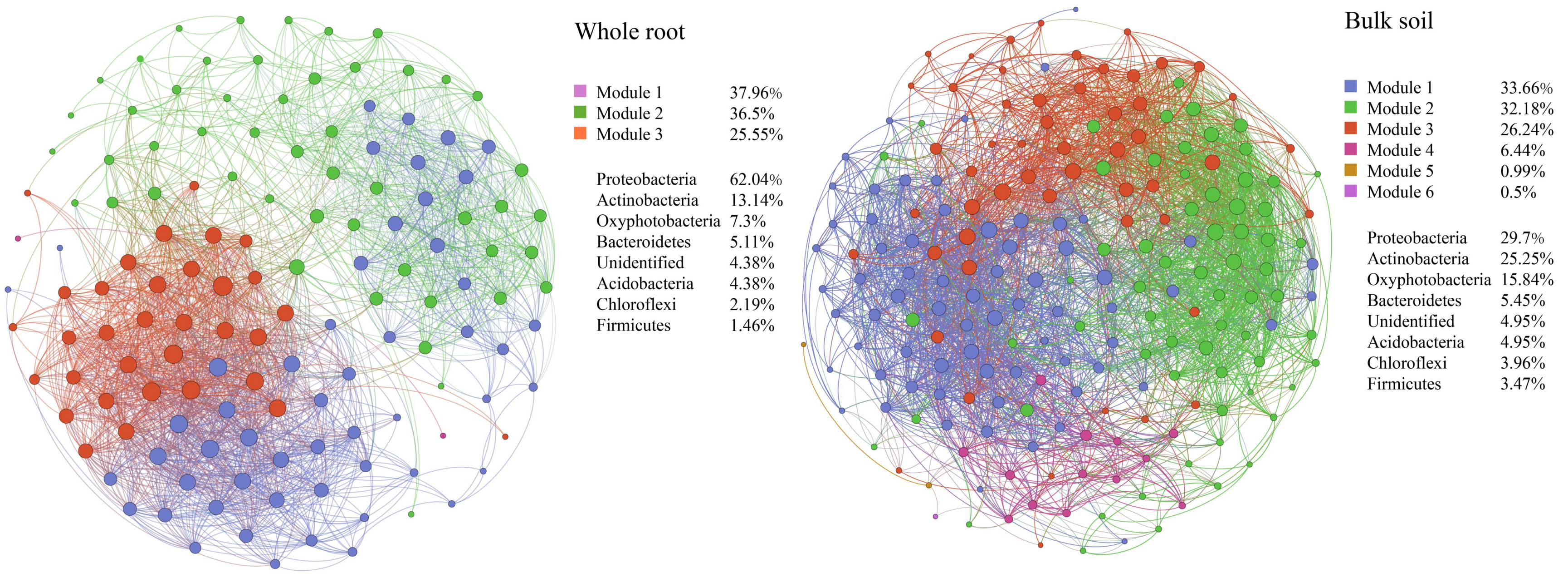

	Empirical networks									Random networks	
	Nodes	Links	PL	NL	N/P	MD	ACC	APL	AD	ACC	APL
Bulk Soil	202	3349	1983	1324	0.67	2.02	0.52	2.15	33.16	0.165±0.002	1.838±0.001
Whole root	137	2064	1662	325	0.20	0.47	0.70	2.20	28.80	0.210±0.003	1.791±0.001

Fig. S9 Co-occurrence networks of the whole root and bulk soil. A connection stands for a strong (Spearman's $\rho > 0.6$) and significant ($P < 0.01$) correlation. The co-occurring networks are colored by module. The size of each node is proportional to the number of connections (i.e. degree). Positive links (PL), negative links (NL), the ratio of negative links to positive links (N/P), modularity (MD), average clustering coefficient (ACC), average path length (APL), average degree (AD).

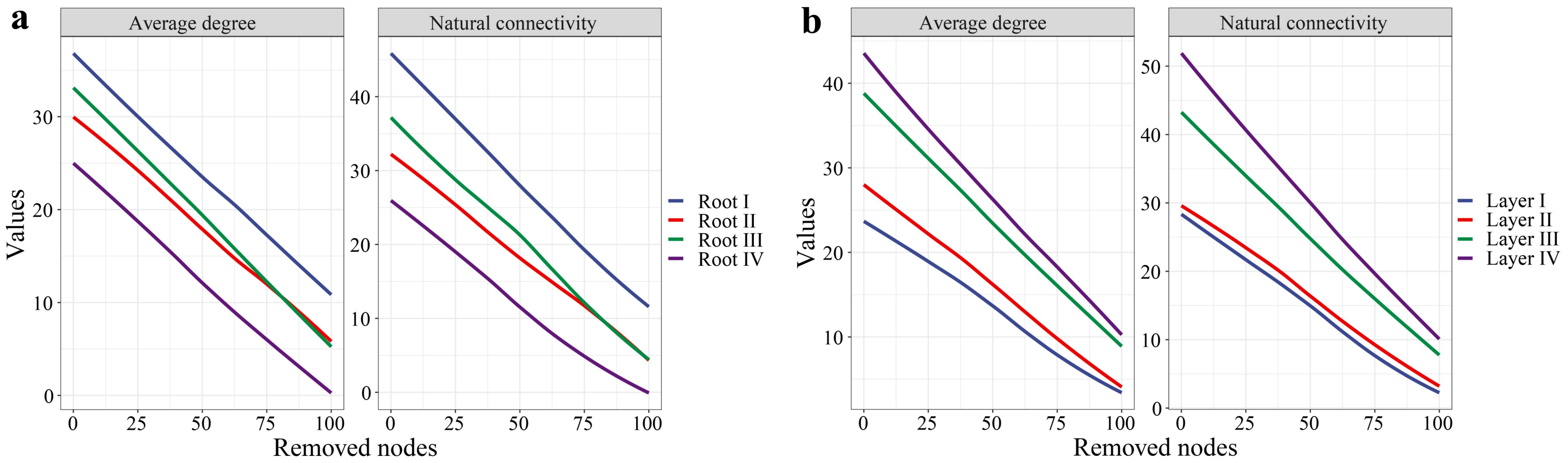

Fig. S10 The robustness of the bacterial network of each root diameter (a) or rooting depth (b).

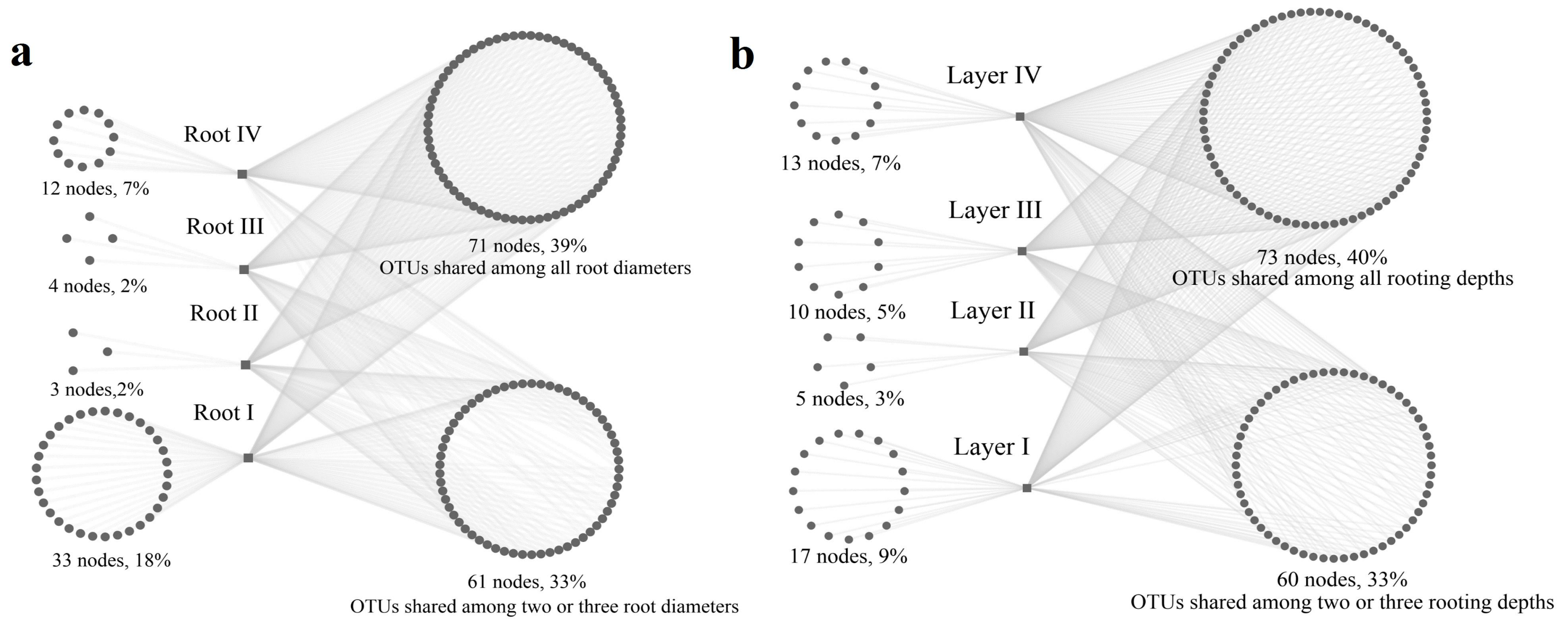

Fig. S11 A operational taxonomic unit (OTU) network map showing the composition and correlation of the OTUs among root diameters (a) and rooting depths(b). OTUs in the left column were unique to one root diameter or rooting depth, while those in the right belonged to multiple root diameters or rooting depths. In total, 71 and 73 OTUs were found across all root diameters or rooting depths, accounting for more than 64.3% and 63.9%, respectively, of all the sequences in the experiment.

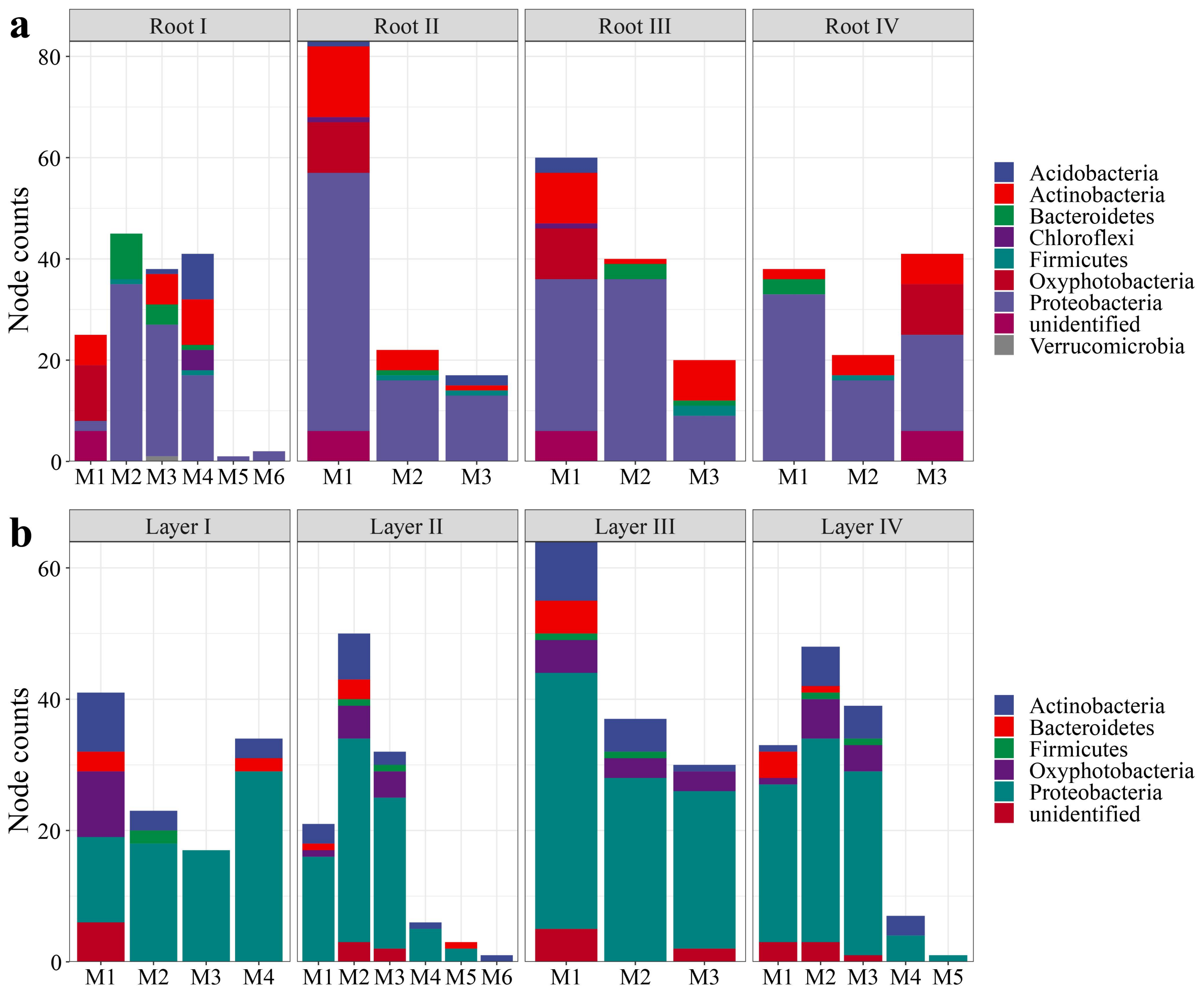

Fig. S12 Bacterial composition of modules in the network of each root diameter (a) or rooting depth (b) at phylum level.
